# Alzheimer’s disease patient groups derived from a multivariate analysis of cognitive test outcomes in the Coalition Against Major Diseases dataset

**DOI:** 10.4155/fsoa-2016-0041

**Published:** 2016-08-19

**Authors:** Inna Tishchenko, Carlos Riveros, Pablo Moscato, Coalition Against Major Diseases

**Affiliations:** 1 Information-Based Medicine Program, Hunter Medical Research Institute, New Lambton Heights, NSW, Australia; 2 School of Electrical Engineering & Computer Science, The University of Newcastle, Callaghan, NSW, Australia; 3 CReDITSS Unit, Hunter Medical Research Institute, New Lambton Heights, NSW, Australia

**Keywords:** Alzheimer’s disease, mini-mental state examination, multivariate analysis

## Abstract

**Aim:**

The mini-mental state examination, commonly used to measure cognitive impairment of Alzheimer’s disease (AD) patients, consists of five test categories. The final score is calculated as their total sum, implying a loss of information.

**Materials & methods:**

In this study, we propose a new multivariate approach to address this issue.

**Results:**

We analyzed the current largest AD-related coalition against major diseases dataset comprising 3717 patients of interest. Our clustering approach revealed five groups of patients associated with distinct characteristics and prognosis. Interestingly, only three cognitive test categories significantly contribute to their determination: registration, attention and recall.

**Conclusion:**

The insight that only these categories are critical for AD group determination may help to resolve the patients’ educational background issue often discussed in relation to the mini-mental state examination assessment.

## Lay abstract

The mini-mental state examination is commonly used to measure cognitive impairment of Alzheimer’s disease (AD)-diagnosed patients. Traditionally, the resulting score of this test is assessed as the total number of correct answers to five types of questions. In this study, we employed a more sophisticated multivariate approach to determine the most common groups of AD patients defined by their cognitive performance in each single category separately. Interestingly, our results revealed that out of the currently employed five question categories only three are crucial for the patient group determination. This insight may impact on the cognitive test design in AD.

**Figure 1.**
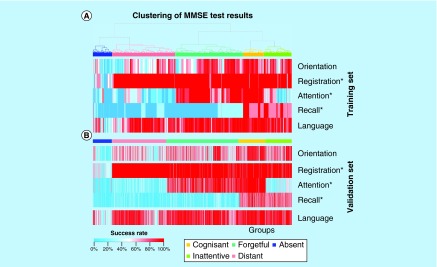
**Heat maps of the training and validation sets.** These heat maps show patients clustered into the groups cognisant (yellow), inattentive (green), forgetful (turquoise), distant (red) and absent (blue), and their corresponding MMSE test results achieved in each category (orientation, registration, attention, recall and language). All scores (denoted as ‘success rate’) were normalized across categories, ranging between 0% (blue) and 100% (red) each, where 0% corresponds to 0 points and 100% to the maximal possible score. The categories significantly differentiating in their success rate values between the patient groups, which were also used for centroids calculation, are denoted with a ‘*’. **(A)** Hierarchical clustering of samples based on the outcomes in five cognitive test categories defines patient groups. **(B)** Patient groups in the validation set assigned using centroids calculated for the categories registration, attention and recall. MMSE: Mini-mental state examination.

**Figure 2.**
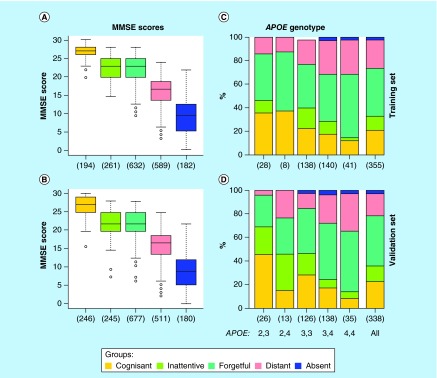
**Association between the patient groups and mini-mental state examination total scores and *APOE* genotypes.** **(A & B)** These box plots show the MMSE total score distributions in the training and validation sets for the five patient groups defined in this study: cognisant (yellow), inattentive (green), forgetful (turquoise), distant (red) and absent (blue). **(C & D)** These two bar charts display the distributions of patient groups stratified by the *APOE* genotype, in the training and validation sets. The last bar corresponds to the global distribution regardless of the *APOE* genotype. Numbers in parenthesis represent the population size of each stratified group. MMSE: Mini-mental state examination.

**Figure 3.**
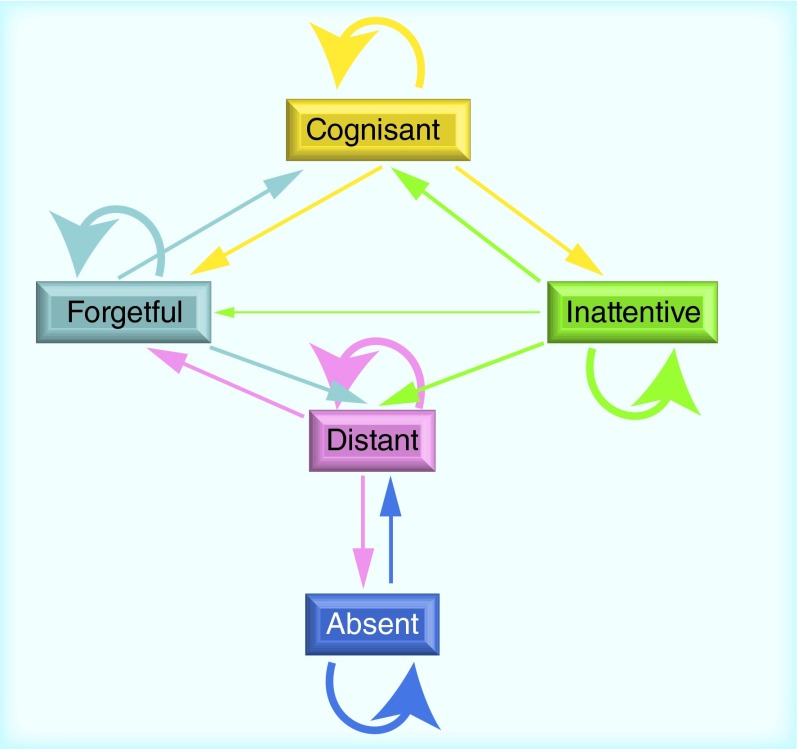
**Transitions across patient groups in the training set.** Patient transitions between the five cognitive groups in the training set are visualized in this graph. The changes were captured using the time frame between six months and four years. The arrow line thickness is proportional to the fraction of people in each transition (the wider the greater is the proportion); only rates greater than 10% are captured in this graph. Arrows leading to the same group stand for patients retaining their status.

Alzheimer’s disease (AD) is the most common form of dementia, accounting for 60–80% of all cases [1]. It is currently considered incurable and it eventually leads to death. The progression of this disease is mostly one-directional with the average survival times after diagnosis lying around 7 years [2], and the probability of living longer than 14 years is smaller than 3% [3].

The recognized main risk factors of AD comprise, but are not limited to, high age, mild cognitive impairment, lack of social engagement, low level of education, family history, *APOE* allele ε4 genotype, and cardiovascular disease and traumatic brain injury [1]. Although the age has been recognized as the strongest risk factor, alone it is not sufficient to cause the disease. A mild cognitive impairment can also be associated with AD, however, it does not always lead to this neurological disease and individuals can return to a normal condition instead [1,4–6]. Multiple studies have previously suggested that social and cognitive engagement supports brain health and leads to a reduced risk of AD [7–9]. Besides these environmental factors, the polymorphic allele ε4 of *APOE*, a major cholesterol carrier that supports injury repair in the brain, has been widely associated with AD, while the ε2 allele is considered to decrease the risk [10–12].

In the course of AD progression, brain synapses start to fail transferring information and neurons eventually die [1]. Consequently, first symptoms of this disease include difficulties in remembering recent events, speaking, writing, planning, solving tasks and a decreased judgment [6]. Advanced stages of AD are associated with confusion, mood swings, depressions, long-term memory losses and disability to reason clearly [13].

Diagnosis of AD is usually based on patients’ cognitive test results, medical and family history, and brain scans [1]. Although the mental state tests alone are not sufficient to determine or diagnose AD, they are essential for the evaluation of the disease progression stage. One of the most commonly applied tests is the 30-point mini-mental state examination (MMSE) questionnaire introduced in 1975 [14–16]. In 2010, a second edition of this questionnaire was released in ten different languages to enable its worldwide application [17]. The standard MMSE test consists of five categories: orientation, registration, attention, recall and language, and the number of maximal points per category is equal to 10, 3, 5, 3 and 9, respectively [14,16,18]. In this test, patients are first asked ten questions related to orientation in time and place. A registration task follows these questions and it includes learning and repetition of three object names. Next, patients are asked to spell backward a five-letter word to test their attention abilities. The recalling skills are determined by patients’ capability to repeat the names of three previously learned objects. To assess patients’ linguistic skills, they are instructed to name shown objects and write down a sentence. The final MMSE score is calculated as the total sum of all points, and it ranges between 0 and 30. Consequently, the test categories orientation, registration, attention, recall and language contribute to this score with the relative weights of 33.3, 10, 16.7, 10 and 30%, respectively. In practice, a total score of 27 or more is considered to represent a normal condition; scores below this threshold are associated with a mild (19–24 points), moderate (10–18 points) or severe (≤9 points) cognitive impairment [19].

Along with the original MMSE version, there are several modifications occasionally applied in practice [20]. One of them incorporates three recall trials instead of one [21]; another version, the ‘26-point telephone MMSE’, has been designed to be conducted over the phone and it skips four questions [22]. The ‘modified mini-mental state (3MS) examination’, on the other hand, has been developed to increase the test sensitivity to orientation, verbal fluency and ability to identify relations between objects [23]. While these cognitive test modifications employ different weights on similar categories, their results interpretation occurs in the same way as for the MMSE, where the total single score is computed as the sum of all points achieved in distinct categories.

Although the current evaluation of cognitive test results using a single score may appear convenient, its interpretation is rather inconclusive with regards to particular cognitive functions. Differences in weights (or the numbers of questions) assigned to single test categories affect the final score calculation; for instance, in the standard MMSE test the orientation skills affect the calculation of the final score the most, while the recalling and registration abilities only provide limited contributions. Educational background has also been previously shown to affect the total MMSE scores, and a revision or adjustment of the current standard MMSE test assessment has been recommended [24–29]. Thus, employing different scoring systems, previously described as test modifications, lead to varying final results incompatible for a direct comparison across test versions and patients.

In this study, we aim at addressing the cognitive test single total score-induced bias by identifying patient groups emerging from a multivariate analysis of the cognitive test results recorded in the current largest AD-related coalition against major diseases (CAMD) database. This project is motivated by the observation that the CAMD database is an aggregation of several trials that employed varying cognitive test versions and are therefore generally incompatible or not directly comparable among each other in terms of the calculated patients’ overall cognitive performance. Thus, our goal is to resolve which test categories contribute to the patient group differentiation the most and to build a model that can be employed to reliably assign a patient to an AD cognitive group associated with certain impairment characteristics and clinical prognosis, independent of the questionnaire version. Furthermore, our objective is to discuss the relation between the determined classification model and patients’ educational background.

## Materials & methods

### Dataset description

Toward achieving the objectives defined above, we analyzed the CAMD dataset based on 6500 AD-diagnosed patients and 24 clinical trials [30]. CAMD was formed in 2008 by the Critical Path Institute, in collaboration with the Engelberg Center for Health Care Reform at the Brookings Institution (Washington, DC, USA). The Coalition brings together patient groups, biopharmaceutical companies and scientists from academia, the US FDA, the EMA, the National Institute of Neurological Disorders and Stroke (NINDS), and the National Institute on Aging (NIA). The data available in the CAMD database have been volunteered by CAMD member companies and nonmember organizations. The use of this dataset for our research was approved by the Human Research Ethics Committee at The University of Newcastle, Australia (approval number: H-2014-0335).

The CAMD dataset contains, among others, patients’ demographic, cognitive test result and *APOE* genotype data, and information about conducted medication trials. The MMSE test records consist of six entries each: five corresponding to the results in questionnaire categories orientation, registration, attention, recall and language, and the sixth to the total score (where a nonstandard MMSE test was applied, the final score was normalized to a 30-points scale). The data preprocessing step, employed to normalize the different clinical trials in the CAMD database, is explained in more detail in Subsection MMSE test results.

For approximately a third of all patients, the information about their *APOE* genotype status is available. These patients are assigned with two alleles, each of the type ε2, ε3 or ε4.

During the medication trials recorded in the CAMD study, patients were given AD-related medications or placebos; intakes of prescribed drugs uncorrelated to AD were also registered. Due to variations among the trials and a wide range of medications, a preprocessing of medication data was necessary to statistically analyze their effect; this procedure is described in Subsection Medications intake.

### Data preprocessing

The original CAMD data were collected by several studies employing varying standards. Therefore, this database required preprocessing, which is described in this section. We would like to emphasize that the procedures outlined in Subsections MMSE test results and Medications intake are specific for this particular database and they were applied to enable a normalization across clinical trials and their merging to a single dataset for our clustering analysis purposes; these steps are not part of the actual data analysis.

#### MMSE test results

In the CAMD dataset, different versions of the MMSE test were applied, under employment of the same test categories, but varying weights. For our clustering purposes, a normalized multivariate dataset is required, and thus, we needed to utilize normalized scores achieved in each of the test categories orientation, registration, attention, recall and language. To that end, we needed to identify the maximal possible scores that could be obtained in each single cognitive test part. Since this information was not included in this dataset, we applied an iterative approach exploiting score distributions to determine them; details on this procedure are provided in Supplementary Data A. Accordingly, we were able to reliably identify three MMSE scoring systems for a total of 3717 patients. One of the versions corresponds to the standard interpretation employing the 30-point scoring system with 10, 3, 5, 3 and 9 points assigned to the categories orientation, registration, attention, recall and language, respectively. Some patients were tested using a 56-point system, with the maximal scores of 20, 6, 10, 6 and 14 in the five categories, respectively. These values correspond to the relative contributions of 35.7, 10.7, 17.9, 10.7 and 25%; this is consistent with the standard version, except the language category is attributed less significance. Another 51-point scoring system, also employed in the CAMD study, is based on the maximal achievable points of 20, 6, 5, 6 and 14 in the same categories, corresponding to the relative weights of 39.2, 11.8, 9.8, 11.8 and 27.5%, respectively. In this version, the categories orientation, registration and recall have a greater impact on the total single score than in the standard. The identification of these weighting schemes was employed to enable a normalization of the results across different trials, as further discussed in Subsection MMSE test results normalization.

#### Medications intake

There are 60,708 records in the medications domain, corresponding to 12,346 different drug IDs, provided for 5996 patients. This is a very sparse data, with a coverage of just 0.082% 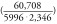. To be able to draw information from this domain, a preprocessing including data merging was necessary. For these purposes, we needed to disregard the intake dosage, time and duration of each drug treatment, although we understand that this information is not insignificant. We applied a recursive approach aiming at clustering together drugs with same generic but different advertized brand names, based on the category tags they share. This led to a total of 132 medication titles. Within this list, we further identified medications recorded as being related to AD treatment: those targeting to treat dementia of AD type (donepezil, galantamine, rivastigmine and memantine), anti-inflammatory medications (ibuprofen, aspirin, celecoxib and naproxen), schizophrenia-related drugs (risperidone and quetiapine), antidepressants (paroxetine, trazodone, sertraline and citalopram), anti-anxiety medications (lorazepam) and cardiovascular conditions-related treatment (clopidogrel and atorvastatin). The vitamins B6, B9, B12, C, E and multivitamins, fish oil and Ginkgo biloba were used as placebos. More details on the original titles of these medications and placebos are provided in Supplementary Data B.

### MMSE test results normalization

Application of the data preprocessing step led to a dataset containing MMSE test results stratified by categories, and the corresponding scoring systems utilized to measure them for each of the 3717 patients. We further used this information to normalize the CAMD dataset across different MMSE test versions, in order to enable the employment of all samples in a single cohort. Thus, we transformed the number of points in each MMSE test category to percentages, with relation to the corresponding maximal possible scores. Accordingly, all normalized values lie between 0 and 100%, where 0% stands for 0 points, and 100% for the maximal score. For example, if each of the patients A and B achieved ten points in the category orientation, where the former patient was examined using the 30-point scoring system (maximal ten points for this test category), and the latter 56-point system (maximal 20 points), then although their absolute scores are the same, the performance of the patient A is 100%, while it is only 50% of the patient B. These are the success rates we considered for further data analysis purposes.

### Clustering of AD patients

In this study, we aim at identifying naturally emerging groups of AD patients based on their multivariate cognitive test results and building a classification model for their determination. Therefore, we first randomly subdivided the cohort comprising 3717 patients into a training (1858 samples) and a validation (1859 samples) set. We then applied a clustering approach to the training set; since the number of features is low (five test categories), we employed the ordinary Euclidean metric to calculate the distance between samples, in accordance with Euclidean distance calculation, Equation 1, where d(p,q) stands for distance between multivariate results of samples p and q, *i* indicates the test category and *N* indicates their total set (five in our case). Each value p and q ranges between 0 and 100%, and thus, the distance values d(p,q) lie between 0% and 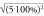
(≈223.6%).

Equation 1
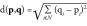


The distance matrix containing Euclidean metric values between all sample pairs in the training set was further employed to compute the Ward hierarchical clustering, where the variance within each group of samples is minimized [31], using R [32]. This calculation led to clusters of patients characterized by mutual cognitive test outcomes; the number of clusters was determined using a trade-off between minimizing this value and maximizing their differentiation from each other.

Since each cognitive MMSE test was conducted and repeated on different dates for same individuals, for the clustering purposes in this study, we utilized records from the first visit date measured before the implementation of medication trials.

### Signature & validation of AD patient groups

Once the patient groups had been established, we aimed at identifying test categories contributing to their separation the most. Thus, we applied the multidimensional rank-based Kruskal–Wallis test (‘one-way ANOVA on ranks’) [33] to each category separately to define its segregation power, using R. The subset of categories with significantly low p-values was further used for centroid calculation for each patient group in the training set; these are the signature vectors containing mean scores of each relevant category for each patient group.

Samples in the validation set, also corresponding to the first cognitive test visit dates, were assigned to one of the patient groups based on their Euclidean distance (**Equation 1**) to the closest centroid calculated above.

### Transitions across patient groups over time

To analyze patient transitions across the groups, we considered the first and last MMSE test results: the former corresponding to any time before or at the beginning of AD medication trials, and the latter at the end or after. Time intervals between the emerging cognitive test results strongly vary across samples with some extending up to 6.5 years. We selected the last visit date to lie within a time period between 6 months and 4 years after the first visit; this choice corresponds to a trade-off between minimizing the time frame between the tests and maximizing the number of samples, while keeping a minimal time lapse between the tests, in accordance with the histogram shown in Supplementary Data C. We found that 1197 samples in the training and 1172 samples in the validation set fulfilled this criterion.

The assignment of patients to a group based on their MMSE test results achieved at the last visit in both training and validation sets was conducted using centroids previously calculated based on the training set (subsection signature & validation of AD patient groups), by means of the Euclidean distance (Equation 1).

### Statistical tests

We computed the percentage agreement and Fleiss’ Kappa κ [34], a statistic measure for assessing the reliability of this agreement, between the patient group labels in the training set emerging from the previously described clustering procedure and those assigned using centroids based on the categories defined to be relevant for these groups segregation. We further computed the same measures for labels assigned in the clustering process and those based on centroids calculated for all five cognitive test categories. The objective of this analysis is to provide insights about the performance of our mathematical model using centroids to represent the resulting patient groups.

We employed the *APOE* genotype information to analyze the association between this marker and patient groups. To that end, we applied the proportion test examining the null hypothesis that the proportions of certain alleles in several groups of patients are the same [35].

We used the medication domain to correlate group transitions over time with certain treatment types. Since AD is generally considered a one-directional disease and patients’ cognitive state generally worsens over time, we compared intake incidence rates of medications in one transition group to those corresponding to a relatively better or worse change. Titles that were ingested by more patients in a transition associated with less severe changes are considered to have a beneficial effect, and vice versa. To calculate significance values of these correlations, we used the binomial test; this is the 2D version of the proportion test. All statistical tests were performed using R [32].

## Results & discussion

### Hierarchical clustering of cognitive test results determines five groups of patients

The clustering approach applied to the training set led to five groups of AD-diagnosed patients, as shown in Figure 1A. For their computation, we employed patients’ success rates ranging between 0 and 100% and representing normalized original absolute scores assigned to each category separately (e.g., a 100% success in the category orientation corresponds to 10 points in the standard MMSE test, while a 50% rate is equal to 5 points in the same test and category). As shown in this figure, the emerging patient groups are associated with divergent incidence rates: cognisant (194 samples), inattentive (261), forgetful (632), distant (589) and absent (182). The relatively small cognisant group is characterized by an overall high performance, particularly in the test categories registration, attention and language; patients in this group only show limited impairment in orientation and recalling abilities. The inattentive group has difficulties with attention and exhibits slight alterations in orientation and recalling skills. The forgetful patients, constituting the largest group, are unable to remember and show slight disorientation. The distant group is characterized by a degradation of patients’ attention and recalling abilities, an impairment in orientation and a slight deterioration of linguistic skills. Patients of the last and smallest group, the absent, are unable to succeed in any of the MMSE test categories.

To examine which categories significantly contribute to the definition of the five patient groups, we applied the Kruskal–Wallis test to each category separately. The categories registration, attention and recall showed a very strong association with the groups stratification (p-values of 4.3 × 10^-302^, 2 × 10^-294^ and 4.7 × 10^-270^, respectively). Although the remaining two categories were still associated with significant results, their log_10_-normalized p-values were almost threefold smaller (p-values of 9.6 × 10^-117^ for orientation and 1 × 10^-100^ for language). Thus, we selected the three categories with the smallest p-values to build a spanning set of features defining the groups, resulting in a reduction of the problem dimensionality from five down to three (categories). It is also visible from Figure 1A that the language and orientation categories do not define the boundaries between the patient groups as accurate as the registration, attention and recall.

Subsequently, we calculated a centroid for each patient group in the training set based on the selected categories registration, attention and recall. The corresponding values are listed in Table 1. These success rates also represent the previous description of each group, where the cognisant patients perform best in all categories, inattentive tend to show little attention, forgetful are not able to recall, distant are only able to register and absent are unable to succeed in any of these categories.

We further compared the assignment of labels using the reduced dimensionality space to those employing all five categories. The labels agreement between the original assignment and those using centroids calculated for the three selected categories (Table 1) is 92.9% (1726 out of 1858 samples) and Fleiss’ Kappa κ is equal to 0.904. An analogous comparison between the patient group labels assigned in the hierarchical clustering process and using centroids calculated based on all five categories led to an agreement value of 90% (1673 out of 1858) and κ equal to 0.866. According to Landis and Koch [36], the greater the κ values the better the agreement, where values greater than 0.81 stand for an ‘almost perfect agreement’. Remarkably, the results in this study do not only confirm the utility of the categories registration, attention and recall for describing the 5D test category space but also demonstrate that they are better representatives. Thus, the five patient groups can be determined in absence of the categories orientation and language, indicating that their supplementary employment along with the three selected test categories does not significantly contribute to the AD group discrimination.

Samples in the validation set were assigned to a cognitive patient group in accordance with the closest centroid from Table 1. The corresponding heat map including values of the two MMSE test categories orientation and language that were not utilized in the appointment process, is shown in Figure 1B. There are 246 cognisant, 245 inattentive, 677 forgetful, 511 distant and 180 absent patients in the validation set. The group sizes and heat maps are consistent across both datasets – an observation that supports the dimensionality reduction down to three categories, and our centroids and groups definition.

### Patient groups are associated with MMSE total score & *APOE* genotype

To visualize the relationship between patients’ original total MMSE scores and their stratification into the five groups defined above, we generated box plots, as shown in Figure 2A & B for the training and validation sets, respectively. Both datasets show coherent results, where the cognisant patients achieve the most (mean: 26.9 and 26.7 points, respectively) and absent the least scores (mean: 10 and 9.3 points, respectively). The inattentive and forgetful patients share the same mean score of 22.4 points in the training set, and 22 and 21.9 in the validation set. Patients from the distant group achieve on average 16.4 points in both datasets, and thus on the MMSE scores scale, they are located between the absent and all other groups. Very low p-values (9.7 × 10^-255^ in the training and 4.2 × 10^-242^ in the validation set) reflect the high significance of the separation between the MMSE score distributions across the patient groups defined above; this is also visible from the first and third quartiles of each box plot with no overlaps between them (except the inattentive and forgetful groups with virtually the same scores). Score ranges between the whiskers, however, are large and substantially intersect each other. This means that although there is a clear association between the groups identified in this study and their total MMSE scores originally calculated as the sum of points achieved in all five test categories, these definitions are not the same and there is a substantial part of patients for which this association is void.

We further analyzed the *APOE* genotypes, a marker widely associated with AD, in relation to the patient groups defined in this study. The resulting bar charts are shown in Figure 2C & D; each bar represents a genotype (*APOE* ε2/ε3, ε2/ε4, ε3/ε3, ε3/ε4 or ε4/ε4) with the corresponding incidence rates. Interestingly, among the AD-diagnosed patients analyzed in this study, the genotype *APOE* ε2/ε2 is not represented at all, and ε3/ε4 is the most common form of *APOE*; this prevalence rates are divergent from those of a general population [10,37]. As shown in the figure, the genotype *APOE* ε2/ε3 contains the largest proportion of cognisant patients, while ε4/ε4 is the least, in both datasets. Generally, the proportions of cognisant, inattentive and forgetful patients decrease, and the ratios of distant samples increment, in the following order: ε2/ε3, ε3/ε3, ε3/ε4, ε4/ε4. Group distributions corresponding to the genotype ε2/ε4 are based on a small number of observations, and thus they are not very reliable; the same holds for the distant group. Since *APOE* ε3/ε4 and ε4/ε4 genotypes can be associated with patients showing poor performance in the MMSE tests, we compared the incidence rates of these two genotypes combined together across our patient groups. The proportion test p-values were found to be small (0.06 in the training and 9.9 × 10^-6^ in the validation set, computed based on 355 and 338 samples, respectively), suggesting that the difference in genotype distributions is significant. The cognisant and inattentive patients were found to contain the smallest rates of *APOE* ε3/ε4 or ε4/ε4, and the distant the highest. The absent group also holds a great proportion of these two genotypes, however, the population sizes are too small (eight and nine samples in the training and validation sets, respectively) to draw a conclusion. Summarizing, the *APOE* genotypes show a certain association with the five patient groups defined in this study, however, this relation is rather weak and alone is not enough to predict or determine the patient groups. Instead, the *APOE* could be interpreted as a factor supporting or opposing patients’ cognitive impairment.

### Consistent transitions across patient groups

Transitions across patient groups over a time frame between 6 months and 4 years are captured in Tables 2 & 3 for the training and validation sets, respectively. Both sets show a remarkable similarity with regards to the relative changes between the groups (denoted in parenthesis). A visualization of these changes over time in the training set is additionally shown in Figure 3. This figure can be interpreted as the evolution of patients diagnosed with AD, generally undergoing a medication or placebo trial, during the time period of up to 4 years.

Most patients tend to retain their group association labels within 4 years. Nevertheless, there are differences in transition behavior between the five patient groups. For instance, the forgetful, distant and absent patients mostly keep their status (patient proportions of 52, 57 and 58% in the training set, respectively), while the majority of the inattentive transit to other groups (only 38% of these patients remain in the same group in the training set). The absent group corresponding to the worst performance in all MMSE test categories only shows substantial changes toward the distant (28%), indicating a slight improvement of the cognitive state in terms of the registration abilities. The distant patients, on the other hand, show some transitions into the forgetful (15% of these patients improve with regards to attention) or absent (18% of these patients deteriorate with respect to registration) groups. Some of the forgetful patients become cognisant again (15%) or decline to distant (20%), which is associated with a loss of the capacity for attention. A substantial part of the cognisant patients changes their label to either forgetful (24%) or inattentive (25%), both representing a slight decline: one with respect to the recall and the other one to the attention abilities. Approximately a fifth of the inattentive patients become cognisant again (19%), and a quarter deteriorate to distant by additionally losing the capability for recalling (25%). Although the forgetful and inattentive groups share virtually the same MMSE total scores, they are rather related to each other through their origin from the cognisant group and a common deterioration to the distant; they show only little direct connection.

We also examined whether these transitions can be linked to the *APOE* genotypes. Thus, we applied the proportion test to genotype distributions with regards to the transition groups discussed above. We found no significance in differentiation between these markers; however, these results may be inconclusive due to small population sizes.

### Medications associated with transitions across groups

We further analyzed transitions between patient groups within the time frame between 6 months and 4 years on their possible associations with the treatment recorded in the CAMD dataset. From our processed medication list, only three titles were found to be significantly (p-values below 0.05) associated with changes in both the training and validation sets: multivitamins (originally classified as placebo), citalopram (an antidepressant) and ginkgo biloba (placebo). An overview of these correlations is shown in Table 4 and more details are provided in Supplementary Data D.

According to these results, the cognisant patients remaining in the same group for up to 4 years are significantly associated with increased intake incidence rates of multivitamins, when compared with patients transitioning from this group to all others combined together. This indicates that these nutrients may have a positive effect and potentially support the AD-diagnosed patients in keeping their cognisant status, since a transition from this group to any other group can be considered as a cognitive deterioration. Recent studies on the vitamins B family, C, D and E also support this finding by emphasizing that they help maintaining a healthy neuronal population and delay brain aging associated with the onset and mild AD [38–42].

A deterioration from the inattentive group toward absent is significantly correlated with increased intake incidence rates of citalopram. Thus, this medication is considered to have an adverse effect, since the absent group is considered to represent a worse cognitive impairment state than inattentive. Previous studies have also shown that although citalopram helps reducing agitation in AD, its practical application is limited by an induced patients’ cognitive decline [43].

Our results further demonstrate that Ginkgo biloba may have a negative effect on distant patients, due to its increased intake incidence rates being associated with transition to the absent group. Its efficacy has been discussed in the literature before, but remains elusive [44–46]. It is also possible that divergent outcomes could be linked to varying mental states of AD patients ingesting this medicinal herb.

Although we were able to depict several associations between the group transitions and medication intakes, many drug titles could not be significantly correlated to a cognitive state improvement or deterioration due to data sparsity. Furthermore, we would like to emphasize that the results of this analysis are only indicative, due to the lack of consistency in the medication trials comprising treatments of varying types and lengths.

## Conclusion & implications

### Patient groups: cognitive subtypes or disease progression?

Clustering of patients based on their cognitive test results in the categories orientation, registration, attention, recall and language, led to an identification of five naturally emerging groups: cognisant, inattentive, forgetful, distant and absent. These patient clusters showed distinct and consistent characteristics in terms of the population sizes and total MMSE scores, and transitions across them indicated that these entities can be associated with varying prognosis. It is inconclusive, however, whether these patient groups represent intrinsic cognitive subtypes of AD or disease progression.

Patient groups defined through the naturally emerging clusters may be interpreted as the representatives for most common and varying multivariate cognitive MMSE test outcomes. This constellation alone does not imply that there are no continuous changes between these groups; it is possible that the clusters themselves could express a progression from one to another. Nevertheless, the varying cluster sizes indicate that there are underlying differences in the occurrence rates within the AD-diagnosed patients: the cognisant and absent patients are substantially less common than inattentive and distant.

In terms of progression paths, there are at least two possible alternatives, also evident in Figure 3: first, from cognisant via forgetful and subsequently distant to absent and second, from cognisant via inattentive and subsequently distant to absent. In the first path, patients lose the ability to remember (forgetful), followed by an additional incapacity for attention (distant) and a decline to the final stage where they are unable to register events (absent). The second path is delineated by loss of patients’ attention (inattentive), followed by an additional incapability for recall (distant) and a further deterioration denoted by the loss of registration skills (absent). Remarkably, following this hypothesis, the forgetful and inattentive patient groups represent different paths with no direct connections between each other (they can be linked to each other using the passage through the cognisant or distant states).

Under consideration of AD being a progressive disease delineated by continuously declining patients’ cognitive states, we suggest the perception of each alternative path described above as a disease progression course from the best to the worst phase, where the most common phases are depicted by our patient clusters. The groups forgetful and inattentive, however, could be considered as cognitive subtypes of this disease. Remarkably, both of these groups show virtually the same MMSE total scores and thus are undetectable using the commonly employed total score as the sum of all points achieved in the five test categories.

### MMSE test categories space reduction & patients’ educational background

Along with the five patient groups, we also identified three MMSE test categories, out of five, mostly contributing to their separation: registration, attention and recall. These are the features that are also sufficient to represent the patient groups. Interestingly, the three selected cognitive test categories can be regarded as examination of basic skills unrelated to further qualifications including the language, which is affected by the education level variations across patients the most. These insights may lead to an improvement of the cognitive test design and also resolve the issue of the educational background, which is currently leading to varying test standards in attempt to adjust the resulting total MMSE scores.

### Translation into practice

The results of our study suggest that the currently employed cognitive test categories registration, attention and recall can be used to stratify AD-diagnosed patients into five groups: cognisant, inattentive, forgetful, distant and absent. This is with the purpose of defining the disease progression state and providing prognosis for patients based on data listed in Tables 2 & 3 and visualized in Figure 3. Following the requisite for translation of our methodology into practice, we describe how the centroids from Table 1 can be applied in clinics in more details in Supplementary Data E, and we provide an Excel file with an implemented calculator in Supplementary Data F & Supplementary Table 1.

### Implications & future outlook

By conducting a multivariate analysis of AD-diagnosed patients, we revealed new insights about the most common AD patient groups and associated relevant cognitive test categories (section ‘Results & discussion’); we also built a model to assign each AD patient to one of these groups in clinics (Table 1 & Supplementary Data F). While the naturally emerged advantage of our centroids is the absence of the categories orientation and language, which are affected by patients’ varying educational backgrounds the most, the main benefit of this application is its independence of the single weights assigned to each test category. This means that any nonstandard cognitive test containing the categories registration, attention and recall can be employed to examine patients’ state, while the interpretation of the results will remain the same. This allows an immediate prognosis in accordance with the results obtained in this study, and a direct comparison between patients across different studies, unbiased by the cognitive space reduction from five down to one single total MMSE score.

We discussed in subsection patient groups: cognitive subtypes or disease progression? that the forgetful and inattentive patients may represent divergent progression paths. A further investigation is required to exclude a bias arising from a possible misdiagnosis and provide more understanding about their underlying nature.

The analysis of the scarce treatment data (subsection medications associated with transitions across groups) pointed to the beneficial effects of multivitamins for the cognisant group. Citalopram showed an adverse effect on the inattentive and Ginkgo biloba on the distant patients, where increased intake incidence rates are associated with a deterioration to absent. We found no significant improvement, however, in individuals administered with the remaining AD-specific drugs or placebos assayed in the CAMD trials. While this result may be biased by the sparsity of the numbers of observations and variations in prescription details, it still shows that AD remains an incurable one-directional disease, where the most positive transitions associated with a treatment are represented by patients retaining their status for up to 4 years without declining.

## Future perspective

We believe that in 5–10 years’ time, the multivariate analysis approaches will take overhand in clinical applications, including the AD impairment state assessment procedure. Thus, we believe that our results may represent the first steps toward this goal, where we demonstrate that AD patients may be stratified into five distinct groups based on their multivariate cognitive test outcomes. More importantly, our analysis led to the conclusion that the category language is not required for these patient groups determination, given that the categories registration, attention and recall are measured. Thus, an exclusion of this part of the test may lead to an improvement of the MMSE test interpretation in the future, since this category is potentially affected by patients’ educational background the most.

**Table 1.** **Centroids calculated in the training set.**

**Category/patient group**	**Cognisant (%)**	**Inattentive (%)**	**Forgetful (%)**	**Distant (%)**	**Absent (%)**
Registration	100	93.3	93.3	100	43.3
Attention	98	44	94	21	18
Recall	83.3	83.3	13.3	6.7	0

Mean success rate values ranging between 0 and 100% in the mini-mental state examination test categories, registration,attention and recall, calculated for the patient groups, cognisant, inattentive, forgetful, distant and absent in the training set.

**Table 2.** **Transitions across patient groups in the training set.**

**First/last**	**Cognisant**	**Inattentive**	**Forgetful**	**Distant**	**Absent**	**Overall**
Cognisant	60 (43)	35 (25)	33 (24)	9 (7)	1 (1)	138
Inattentive	33 (19)	68 (38)	22 (12)	44 (25)	10 (6)	177
Forgetful	65 (15)	28 (7)	218 (52)	86 (20)	24 (6)	421
Distant	13 (3)	25 (7)	57 (15)	218 (57)	67 (18)	380
Absent	1 (1)	4 (5)	6 (7)	23 (28)	47 (58)	81
Overall	172	160	336	380	149	1197

The absolute and relative numbers of patients changing their group label within a time frame between 6 months and 4 years in the training set are listed in this table. Labels assigned to samples based on the MMSE test results achieved at the first visit are represented by rows, and those at the last by columns. Numbers in parenthesis represent the relative number of patients (proportion) involved in each transition, calculated with respect to the whole population corresponding to the label determined at the first visit (last column). The population size of each transition group used for this analysis is listed in the last column for the first visit and in the last row for the last visit.

**Table 3.** **Transitions across patient groups in the validation set.**

**First/last**	**Cognisant**	**Inattentive**	**Forgetful**	**Distant**	**Absent**	**Overall**
Cognisant	76 (44)	53 (31)	30 (17)	9 (5)	6 (3)	174
Inattentive	35 (20)	56 (32)	19 (11)	48 (27)	18 (10)	176
Forgetful	63 (15)	28 (7)	225 (52)	89 (21)	25 (6)	430
Distant	18 (3)	23 (7)	35 (11)	191 (60)	59 (19)	316
Absent	1 (1)	5 (7)	5 (7)	25 (33)	40 (53)	76
Overall	183	165	314	362	148	1172

The absolute and relative numbers of patients changing their group label within a time frame between 6 months and 4 years in the validation set are listed in this table. Labels assigned to samples based on the MMSE test results achieved at the first visit are represented by rows, and those at the last by columns. Numbers in parenthesis represent the relative number of patients (proportion) involved in each transition, calculated with respect to the whole population corresponding to the label determined at the first visit (last column). The population size of each transition group used for this analysis is listed in the last column for the first visit and in the last row for the last visit.

**Table 4.** **Details on medications associated with transitions across patient groups.**

**From**	**To**	**Treatment**	**Effect**	**p-value (T)**	**p-value (V)**
Cognisant	Cognisant	Multivitamins	Beneficial	0.031	0.025
Inattentive	Absent	Citalopram	Adverse	0.0056	0.031
Distant	Absent	Ginkgo biloba	Adverse	0.019	0.046

Significant associations between increased medication intake incidence rates and transitions between patient groups (‘From’ and ‘To’) over the time frame of up to 4 years are listed in this table. Medication titles are listed in the column ‘Treatment’. ‘Effect’ indicates the relative impact of the corresponding drug on the original patient group depicted in the column ‘From’; ‘beneficial’ means that the intake of a particular drug listed in ‘Treatment’ is associated with relatively positive changes for the patient group listed in the column ‘From’, and ‘adverse’ stands for a negative effect and indicates that the corresponding treatment should be prescribed with caution for patients in the group listed in the column ‘From’. The Binomial test p-values are recorded in the last two columns for the training (‘T’) and validation (‘V’) datasets, respectively; more details on the calculation of these values are provided in Supplementary Data D.

**Executive summary**

The mini-mental state examination (MMSE) is a questionnaire employed to assess the Alzheimer’s disease (AD)-diagnosed patients’ mental state; it consists of five test categories: orientation, registration, attention, recall and language.
Evaluation of the MMSE outcomes by means of a single total score is biased by the patients’ educational background and varying test versions employing the same categories, but different relative weight attributions.
The multivariate analysis on cognitive test outcomes conducted in this study led to an identification of five groups of AD patients: cognisant, inattentive, forgetful, distant and absent.
These patient groups are associated with distinct characteristics and prognostics.
Out of the five MMSE questionnaire categories, only three were found to be critical for these patient groups determination: registration, attention and recall.
The mathematical model (centroids) proposed in this study for identification and characterization of the AD patient groups is robust, it may help resolving the patients’ educational background bias and it allows a direct comparison across patients independent of the cognitive test version (provided it contains the categories registration, attention and recall).
Multivitamins may support cognisant AD-diagnosed patients with a mild recalling impairment.
