# Supplementary material for: Alzheimer’s disease patient groups derived from a multivariate analysis of cognitive test outcomes in the Coalition Against Major Diseases dataset
Source: Future Sci OA. 2016 Aug 19;2(3):FSO140. doi: 10.4155/fsoa-2016-0041 (PMC5137968; doi:10.4155/fsoa-2016-0041)
Supplement: Supplementary file 1 [file fsoa-02-140-s1.docx]

Supplementary Data

1. MMSE Data Pre-Processing

In order to determine the maximal scores that could be achieved in each category in a single questionnaire, we applied an iterative approach exploiting the initial assumption that each of the 24 individual study groups in the CAMD database applied the same MMSE scoring system, generally divergent across these studies. Accordingly, we executed the following steps for each of the 24 trials separately:

1. Obtain a histogram for each category based on all patients in this trial.
2. Based on the limit values, draw an inference on the maximal number of points per category (scoring system), and compute a normalized total MMSE score based on these values.
3. Compare the computed and provided MMSE scores to test whether the inferred scoring system is valid for all patients in the trial.
4. Segregate samples with incongruent MMSE scores and consider them a different trial.
5. Repeat the procedure from item 1 until there are no inconsistent cases present.

We could reliably identify three MMSE scoring systems for a total of 3,717 patients. One of the versions corresponds to the standard interpretation employing the 30-point scoring system with 10, 3, 5, 3 and 9 points assigned to the categories *orientation*, *registration*, *attention*, *recall* and *language*, respectively. This system is used in the individual studies labelled as “1000” and “DON” in the CAMD data set, and accounts for a total of 392 samples (102 and 290 respectively). Histograms visualizing the achieved scores distributions are shown in Figures S1 and S2. Another version employs a 56-point scoring system, with the maximal scores of 20, 6, 10, 6 and 14 in the five categories, respectively. The studies labelled as “1013”, “1014” and “A000”, and a part of the “E202” study were found to use this modified scoring scheme. This accounts for a total of 2,729 samples (719, 644, 326 and 1,040, respectively); the corresponding histograms are plotted in Figures S3, S4, S5 and S6. Finally, the other part of the “E202” study was found to be based on a 51-point scoring system, with maximal achievable points of 20, 6, 5, 6 and 14 in the categories *orientation*, *registration*, *attention*, *recall* and *language*, respectively. A total of 596 samples was found to exploit this system; the corresponding histograms are provided in Figure S7.


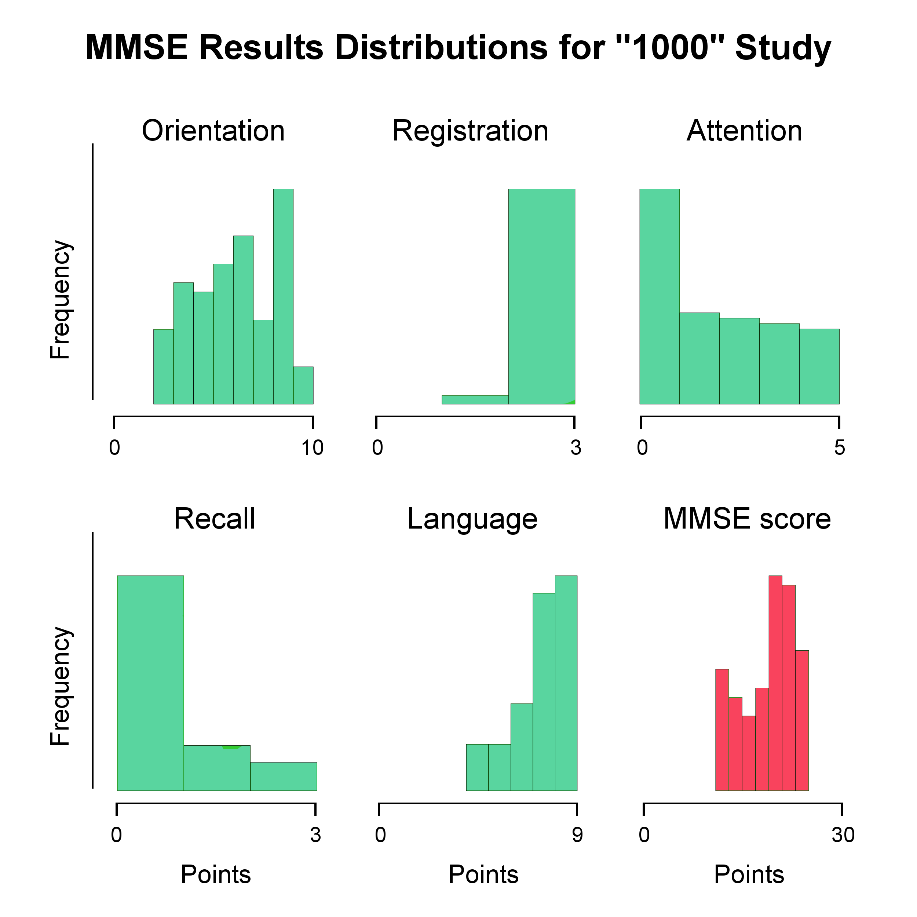


*Figure S1*. **MMSE-test results distributions for the “1000” study.**


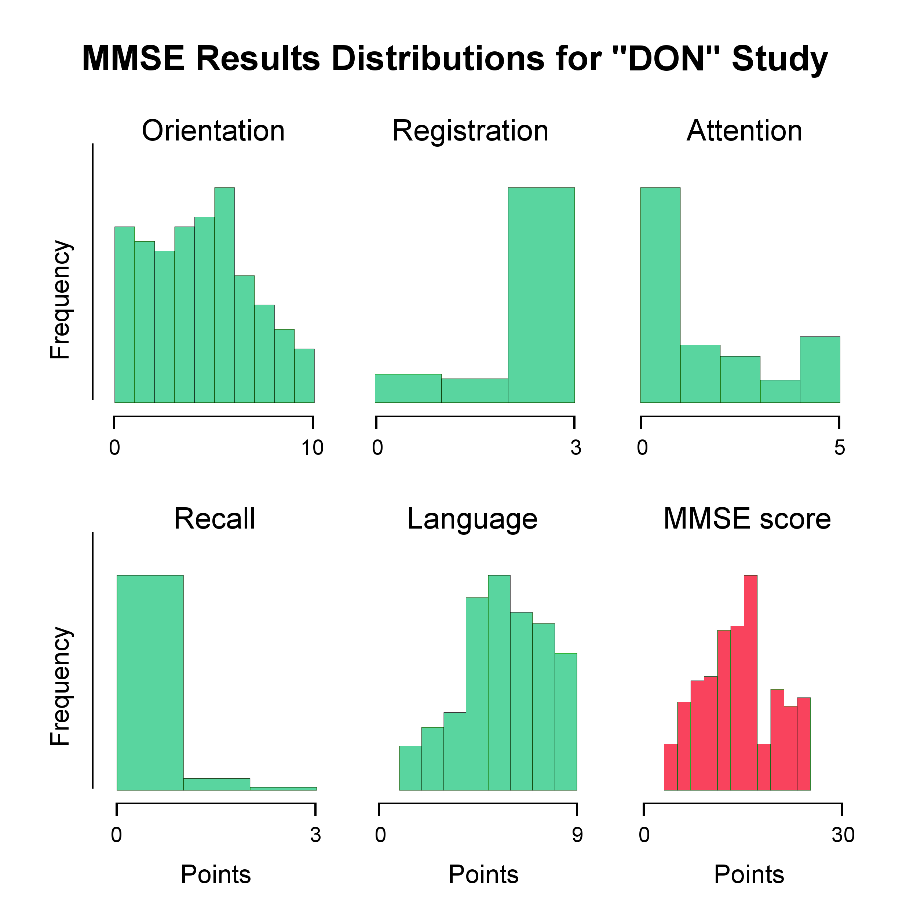


*Figure S2*. **MMSE-test results distributions for the “DON” study.**


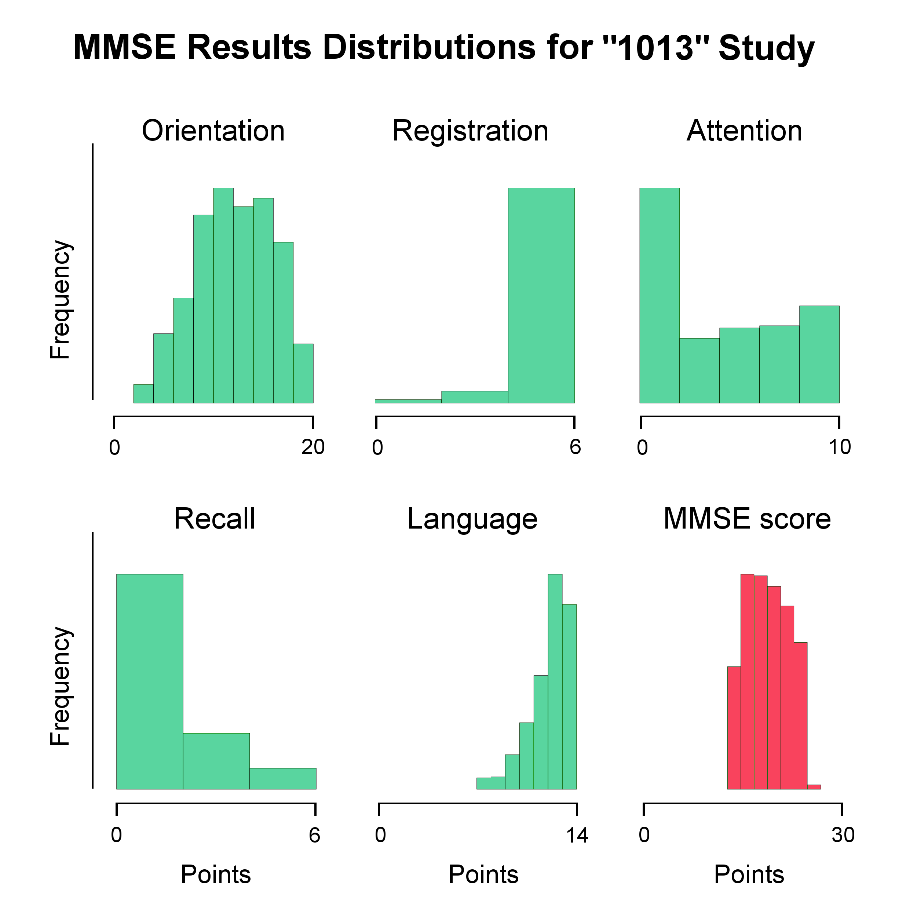


*Figure S3*. **MMSE-test results distributions for the “1013” study.**


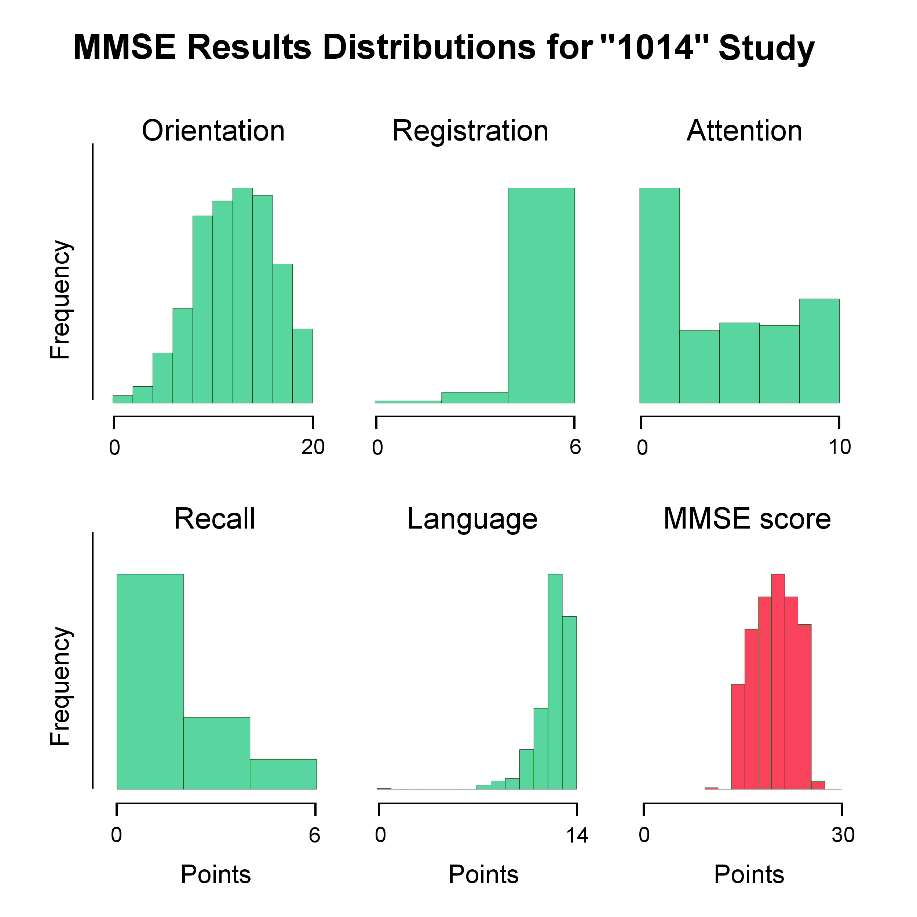


*Figure S4*. **MMSE-test results distributions for the “1014” study.**

**
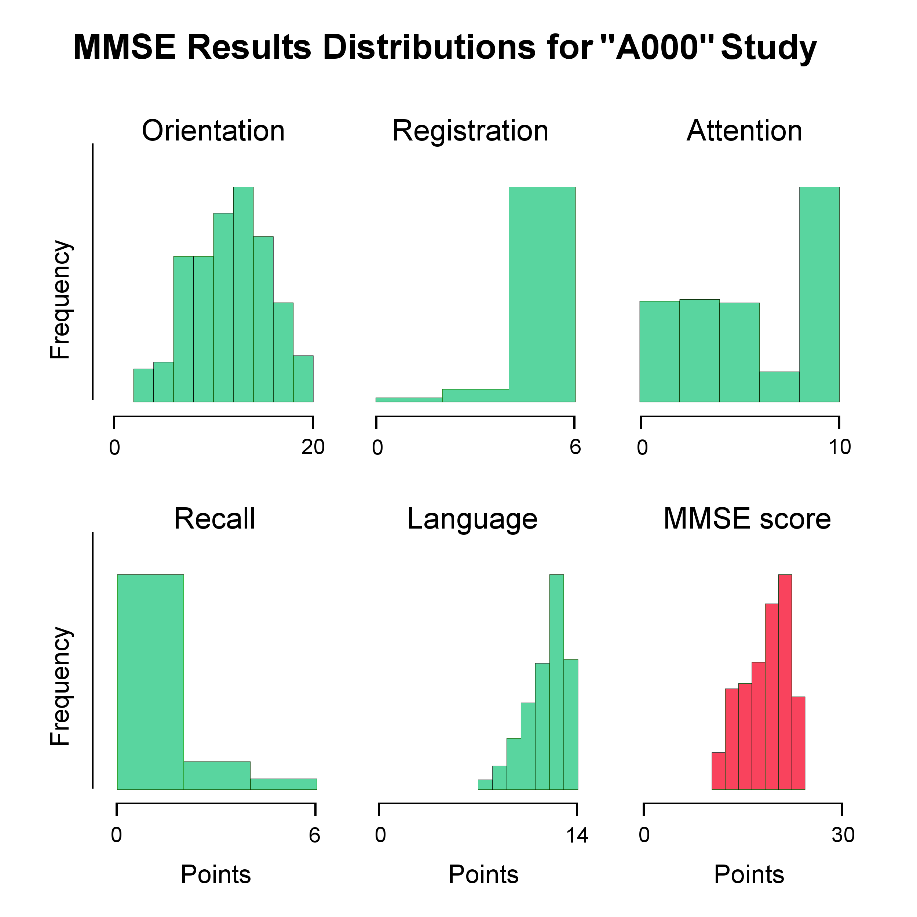
**

*Figure S5*. **MMSE-test results distributions for the “A000” study.**

**
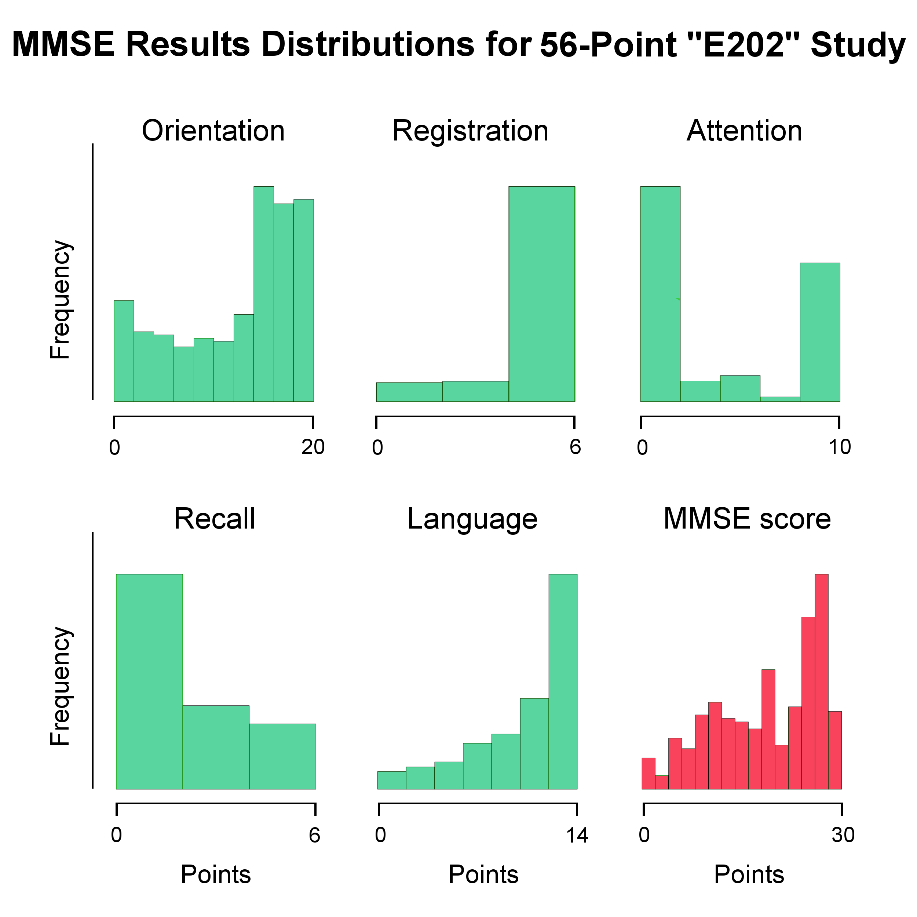
**

*Figure S6*. **MMSE-test results distributions for the 56-point-based “E202” study.**

**
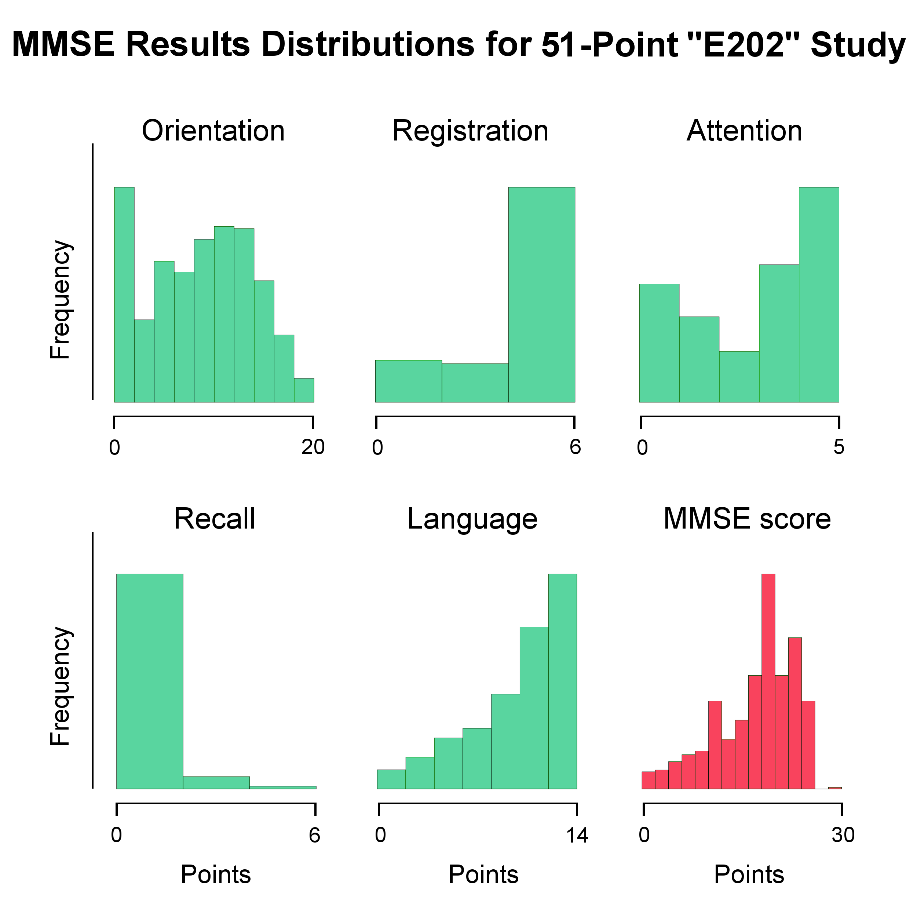
**

*Figure S7*. **MMSE-test results distributions for the 51-point-based “E202” study.**

1. Medication Categories and Classes

The list of medication category and class names from the CAMD data set, aggregated in our study from 12,346 titles, is provided below. It consists of two main classes: AD-related treatment and placebos, in accordance with their classification in the CAMD data set.

## Alzheimer's disease-related treatment

### AD-Related Drugs

"DONEPEZIL"

"ARICEPT", "DONEPEZIL", "ARICEPT / DONEPIZIL", "DONEPEZIL (ARICEPT)", "DONEPIZIL", "DONEZEPIL", "DONEPEZIL HCL", "BONEPEZIL", "ARICEPT 10", "ARICEPT (DONEPEZIL)", "MEMAC", "ARICEPT 10MG", "ARICEPT 10 MG", "MEMAC 10 MG", "DONEPEZIL 10 MG", "ARICEPT 5MG", "MEMAC 10", "ARICEPT (R)", "ARICEPT : DONEPEZIL HCI", "ARICEPT (R) (DONEPEZIL)", "DONEPEZILO", "ARICEPT 5", "ARICEPT TM 10 MG", "DOMEPEZIL", "ARICIPT", "DONNEPEZIL", "ARICEPT OD", "DONEPEZIC HCL", "ARICEPT PO", "ARICEPT DONEPEZIL HCL", "ARRICEPT", "DONEPEZIL / ARICEPT", "DONEPEZIL (T. ARICEPT)", "ARICEPT DONEPEZIL", "T. ARICEPT", "DONEPEZIL HYDROCHLORIDE", "DONEPEZILE", "DONAZEPIL", "DONAPEZIL", "(DONEPEZIL) ARICEPT", "DONEPEZIL (COMMERCIAL)", "COMMERCIAL DONEPEZIL", DONEPEZILHYDROCHLORIDE", "ARICEPT(DONEPEZIL)", "ARIZEPT", "ARICEPT 5 MG", "COGITON", "YASNAL", "DONEPEZIL (COGITON)", "DONEPEZIL(YASNAL)", "DONEPEX", "YASNAL /DONEPEZIL/", "ARICEPT /DONEPEZIL/", "DONEPZIL (ARICEPT)", "ARICEPT (DONEPEZIL 10MG)", "DONEPEZIL(ARICEPT)", "ARICEPT-DONEPEZIL", "DONEPIZIL/ARICEPT", "ARIZEPT 10", "ARICEPT TBL.", "DONEPEZIL 5 MG", "ARICEPT 10 MG.", "DONECEPEZIL", "ARICEPT (DONEZEPIL)", "MEMEC", "MEMAC 5", "COGITON 5 MG", "CRISTACLAR", "ERANZ", "DONEPECILO", "DONEPECIL", "DAZOLIN (DONEPEZIL)", "DONEPEZIL (ERANZ)", "ARICEP", "TAB DONEPEZIL", "ARICEP (DONEPEZIL)", "ERANZ (DONEPECIL)", "ARICEPT D", "ARICEPTD", "(ARICEPT) DONEPEZIL HYDROCHLORIDE", "ARICEPT= DONEPEZIL HCL", "DONEPEZIL 10MG", "DONEPEZIL 5MG", "ARICEPT 10 MG QAM", "DONEPEZIL 10 MG QD", "DONEPEZIL HCI", "DONEPEZIL HCL (ARICEPT)", "DONEPEZIL 5ML"

"GALANTAMINE"

"RAZAYNE", "REMINYL", "RAZADYNE ER", "RAZADYNE (GALANTAMINE)", "GALANTAMINE", "GALANTAMIN", "GALANTAMINE HYDROBROMIDE", "REMINIL", "REMINYL 24MG", "REMYNIL", "REMINYL RET", "GALANTAMINE BD", "REMINYL : GALANTIMINE", "REMYNIL (R)", "GALANTAMINA", "REMINYL 12MG", "REMINYL 12 MG", "RAZODYNE ER", "REMENYL", "REMINYL ER", "REMINYLER", "REMINYL LA", "REMINYL 12 MG BID", "RAZADYNC", "GALANTAMINE RETARD", "REMINYL RETARD", "NIVALIN", "REMINYL 8 MG", "REMINYL LP", "REMINYL /GALANTAMIN/", "REMINYL 16 MG", "REMINYL ( GALANTAMINA)", "GALANTAMINA ( REMINYL)", "REMINYL 12", "REMINYL XL", "REMINYL(GALANTAMINE)", "REMINYL PR", "RAZADINE", "GALANTAMINE (REMINYL)", "REMINYL GALANTAMINE", "GRALANTAMINE", "REMINYL (GALANTAMINE)", "GALANTAMINE HYDROCHLORIDE", "GALANTOMINE", "GALANTAMINE 12 MG", "GALANTAMINE VS PLACEBO", "GALANTAMINE HBR (REMINYL)", "GALANTAMINE HBR", "REMINYL 4 MG"

"RIVASTIGMINE"

"EXELON", "EXELON PATCH", "RIVASTIGMINE", "RIVASTIGMIN", "EXELON 6MG", "RIVASTIGAMINE", "EXELON 9 MG", "RIVASTIGMINA", "EXELON 4.5 MG", "PROMETAX", "RIVASTIOMINA", "EXELON RIVASTIGMINE", "PROMETOX", "PROMETAX (R) (RIVASTIGMINE)", "EXELON (R)", "PROMETAX TM (RIVASTAGMINE)", "PROMETAX (TM) (RIVASTASMINE)", "PROMETAX (RIVASTAGMINE)", "EXCELON", "RIVISTIGMINE", "EXCELAN", "EXELON 3 MG BID", "EXELON 4.5", "RIVASTIGMINE PATCH", "RIVASTYGMINA(EXELON)", "EXELON(RIVASTYGMINA)", "RIWASTYGMINE", "EXELON /RIVASTIGMINE/", "RIVASTIGMINUM (EXELON)", "EXELON ( RIVASTIGMINA)", "RIVAGSTIMIN", "RIVASTIGMINE (EXELON)", "EXELON TRANSDERMAL PATCH", "EXCELEN PATCH", "EXLELON", "RIVASTIGIME", "RIVASTIGMINE TARTRATE", "RAVISTIGMINE", "EXELON-RIVASTIGMINE", "EXETON", "VIVASTIGMINE", "EXELON = RIVASTIGIMINE TARTRATE", ["EXELON (RIVASTIGMINE)", "RIVASITIGMINE", "RIVASTIGMINE 1.5 BID", "RIVASTIGMINE TARTRATE (EXELON)", "RIVASTIGMINE 6 MG BID", "RIVATIGMINE", "EXELON 6 MG"

"MEMANTINE"

"NAMENDA", "EBIXA", "MEMANTINE", "EBIXA 10", "MEMANTINA", "EBIXA (R)", "AXURA", "AXURA 10 MG", "AXURA 10MG", "EBIXA (TM) (MEMANTINE)", "MAMENDA", "MEMANTINE HYDROCHLORIDE", "MEMANTIN", "NEMENDA", "MEMATINE", "NAMENDA MEMANTINE", "AXURA/NAMENDA"

### Anti-Inflammatory Medications

"IBUPROFEN"

"ADVIL", "IBUPROFEN", "MOTRIN", "IBUPROPHEN", "BRUFEN", "IBALGIN", "IBUPROFENO", "NUROFEN", "IBOPROFEN", "IBUPRODEN", "MOMENT", "INZA (R) IBUPROFEN", "ESPIDIFEN", "IBUPROFENE", "NEOBRUFEN", "IBUPROFENO 10", "IBUPROFEND", "IBUPROFEN 600", "MOTRIM", "ADVIL PRN", "ADVIL LIQUID GELS", "IBUPOAFEN", "IBUPROFEN 500 MG", "IBUPROFEN (SUPPOSITORY)", "IBUPROFEN (NEUROFEN)", "IBUPOFEN", "IBUROFEN", "IBUHEXAL 400", "IBUPROFRN GEL", "NEOROFEN", "IBUPROFEN 600", "IBUPROFEN PATCH", "DOLORUB GEL (IBUPROFEN)", "IBUTAD", "IBUPROPHIN", "IBUPROFAN", "IBUPROPHAN", "IBUPROFEN 400 MG", "ADVIL 200MG", "EQUATE", "(ADVIL) IBUPROFEN", "IBUPROFEN GENERIC"

"ASPRIN"

"BABY ASPIRIN", "ASPIRIN", "CARDIZEM", "ASPIRIN EC", "DILTIZEM ER", "ASPRIN", "ASAFLOW", "CARDIOASPIRINE", "ASPIRIN PROTECT", "ASA", "ANOPYRIN", "ASS", "GODAMED", "TROMBO ASS", "THROMBO ASS", "ADIRO", "TROMALYT", "CARDIZEM CD", "CARTIA", "ASTRIX", "ASPIRIN (CARTIA)", "DISPRIN", "CARDIPRIN", "DILTIAZEM", "CARTIA (ASPIRIN)", "ENTERIC COATED ASPIRIN", "BOKEY", "ASPIRIN COATED TABLET", "ASPIRIN 100 MG", "ASPIRIN COATED", "DILTIAZEN", "CARDIOASPIRINA", "CARDIOASPIRIN", "ASCRIPTIN", "ASPIRINETTA", "CARDIO ASPIRINA", "CARDIO ASPIRIN", "TILDIEM", "ASPIRINETIA", "ACETILSALICILIC ACID", "ACETYL-SALICYLIC ACID", "ACETOSAL", "ACETYLSALICYLACID", "ACETYLSALICYL ACID", "ACETYLSAL. ACID", "ACETYL SALICYL-ACID", "ACETYL SALICYL", "ACETYL SALIC ACID (ASA)", "ACETYLSALICYLIC ACID", "ACETYLSAL ACID", "ACETYLSALACID", "ACETYLSAL", "ACETYLSALIC ACID", "ACETYLSALICYL", "ASPEC 75", "ASPEC", "DILZEM HCL", "DISPRIN (R)", "DISPRIN (ASPIRIN)", "SOL ASPRIN DISPRIN", "ECOTRIN", "ZILDEM", "DISPRIN CV", "CALCICARD", "TILAZEM", "ASPIRINA", "ACETILSALICYLIC ACID", "ACETIL SALICYLIC ACID", "BIOPLAK", "ASPIRINE", "ACETILSALICILYC ACID", "ADIRO 100", "ADIRO 100 MG", "TROMALYT 150", "MASDIL RETARD", "DILTIAZOM", "ACETYL SALICYLIC ACID", "CARTIA XL", "CARTIA XT", "CARDIZAM", "DILTIAZEM XT", "(PROPHYLAXIS) ASPIRIN", "NOVASEN", "ASAPHEN", "ENTROPHEN", "NOVO DILTIAZEM", "RIVASA", "ENTERIC COATED ASA", "ASA OD", "EENTERIC COATED ASPIRIN", "ENTERIC COATED ACETYLSALICYLIC ACID", "EC-ASPIRIN", "EC-ASA", "ASA EC", "ASA/NOVASEN", "EC ASA", "ECASA", "ASA ENTERIC", "DILTIAZEM ER", "BAYER ASPIRIN", "DILITIAZEM", "DILTIAZEM XR", "CARDIA XT", "ENTEREC COATED ASPIRIN", "ENTCREL COATED ASPIRIN", "DILTIZEM HCL", "ST JOSEPH'S ASPIRIN", "CARDIZEM XR", "ECOTRIL", "TIAZAC", "BABY ASA", "ASPINN", "ASPIRIN ASA", "ENTERIC ASPIRIN", "CARDIZEM LA", "CHILDREN'S ASPIRIN", "DILTAZEM", "ASPIRIN (ASA)", "ASS ACETYLSALICYLACID", "AAS", "ACETYLICSALICYLIC ACID", "ACID ACETYLSALICYLIC", "ENTERIC - COATED ASPIRIN", "ASS (ASPIRIN)", "(ASPIRIN) ECOTRIN", "DILTIAZEM HCL", "DISPIRIN", "ACETYLIC SALICYLIC ACID", "ACETYLSALYCYL ACID", "DILTIAZEM LA", "ASPIRIN E/C", "DILITAZEM", "ACIDUM ACETYLSALICYLICUM", "PRIMASPAN", "HERZ ASS", "ACETYLSALIZYLSÃ¯Â¿Â¤URE", "ACETUL SALICYLATE", "ACARD", "ACIDUM ACETOLOSALICYL.", "ACETYLOSALICYLIC ACID", "ANOPYRIN /ACIDUM ACETYLSALICYLICUM/", "ASPIRIN 100", "TROMBYL", "ASPIRIN DISPERSIBLE", "ASTRIX 100", "ASTRIX(ASPIRIN)", "ASPIRIN PROTECT (ASPIRIN)", "ASADOL", "BUFFERIN", "THROMBO-ASS", "DILTIAZEM RETARD", "THROBO-ASS", "THROMBOASS", "MONOTILDIEM 200 MG LP", "ASS 100", "ACETYLSALYCILSÃ¯Â¿Â¤URE (ASS)", "ASS100", "SALOSPIR", "CARDIOASPIRIN 100", "POLOCARD", "ECOSPRIN", "ACETIL SALISILIC ACID", "A.S.A.", "BABY ASPRIN", "TAZTIA XT", "ASCRIPTON", "BAYASPIRIN", "BITILDIEM", "HJERTEMAGNYLI", "ASS CARDIAC PROPHYLAXIS", "ASPIRINCARDIO", "DILTIAZEM(HERBESSER)", "ENTERIC-COATED ASPIRIN", "ACETIL SALICILIC ACID", "CARTIA- ASPIRIN", "ASAPHEN EC", "ACETYLSALICYLIC ACID (ASPIRIN)", "COATED ASPIRIN", "CHILDREN'S BAYER", "ECOTRIN (ASPIRIN)", "EC ASPIRIN", "ASPIRIN ENTERIC COATED", "DILACOR XR", "EC ASPRIN", "ASPRIRN", "SOLUBLE ASPIRIN", "ASRIRIN", "DILTIAZEM HYDROCHLORIDE", "CARDIAZEM CD", "ENTERIC COADED ASPIRIN", "BABY ASPIRIN (SALICYLATE)", "DILTIAZEM SR", "ACETYLSALICYLIC ACID ASPIRIN", "ACETYLSALISILIC ACID", "(ACETYLSALICYLIC ACID)", "ASA ASPIRIN", "(ECOTRIN) ASPIRIN", "DILTIAZEM EXTENDED RELEASE"

"CELCOXIB"

"CELEBREX", "CELEBREX (CELECOXIB)", "CELECOXIB", "COLECOXIB", "CELECOBIX", "SOLEXA", "CELEBRA", "CELCOXIB", "CELECOXID", "(CELEBREX) CELECOXIB", "CELICOXIB"

"NAPROXEN"

"ALEVE", "NAPROXEN", "INZA", "NAPROSYN", "NAPROSYN SR", "MOMENDOL", "SYNFLEX", "MAPROXEN", "NAPOXEN", "ALLEVE", "NAPROSEN", "NAPROXEN SODIUM", "NAPROXEN EC", "T. NAPROXEN", "NAPROXEN (ALEVE)", "NAPROXENO", "ANAPROX", "(NAPROXEN- SODIUM) ALEVE", "NAPROXIN", "ALEVE NAPROXEN SODIUM", "NAPROXYN"

### Schizophrenia-Related Drugs

"RISPERIDONE"

"RISPERIDONE", "RISPERDAL", "RISPERIDON", "RISPERODONE", "RISPERIDONE ON/PRN", "RESPIRIDONE", "RISPERIDONA", "RISPERIDONE (RISPERDAL R)", "RISPERDAL 1 MG", "RISPERDAL 1MG", "RISPERDAL M", "RISPERADOL", "RISPERADAL", "RIPERDAL", "RISPERDOL", "RESPERIOROL", "RESPERIDOL", "RISPERIDAL", "RESPERODAL", "RESPERDAL", "RISPEDROL", "RISPERIDONE (RISPERDOL)", "RISPERDONE", "RISPEN", "RISPOLUX", "RISPERIDONE 0,25 MG", "HUNPERDAL", "RISPOLEPT", "RISPERON 1 MG", "GOVAL (RISPERIDONE)", "RISPERIDONE 1%", "RISPERDOLE", "RISPERIDOL", "RISPERIDONI", "RISPERDAL (HS)", "RESPIRADONE", "RESPARDIL"

"QUETIAPINE"

"SEROQUEL", "QUETIAPINE", "QUETINPINE", "SEROQUEL 75 MG", "SEROQUEL 1/2 CP", "QUETIAPINE (SEROQUEL)", "SEROQUEL 100 MG", "SEROQUIL", "SEROGUEL", "SERROQUEL", "QUETIZPINE 400 MG", "QUETICIPINE FEMARATE", "QUETIAPINE FUMARATE", "SEROQUEL (QUETIAPINE)", "SEROQUEL(QUETIAPINE FUMARATE)", "QUETIAPINA", "QUETIDIN", "QUTIPIN (QUETIAPINE)", "QUETIEPINE", "QUTIAPINE", "ASICOT (QUETIAPINE)", "QUETIAPINE FUMARATO"

### Anti-Depressants

"PAROXETINE"

"PAXIL", "PAXIL CR", "SEROXAT", "PAROXETINA", "AROPAX", "PAROXETINE", "DEROXAT", "SEREUPIN", "PAROXETINA CLORIDRATO", "SEROXOT", "SANDOZ PAROXETINE", "AROPAX (PAROXETINE)", "MOTIVAN", "SEROXAT 20 MG", "SEROXAT 20", "PAROXTINE", "PAROXETINE-HCL", "PAXIL-C-R", "PAROXETINE HCL", "PAROXETINE HYDROCHLORIDE", "PAROXETIN", "SEROXAT PAROXETINE", "PAROXAT 20", "PAXTINE", "SEROXATE", "GENERIC PAXIL", "PAROXETINE 20 MG", "DAPAROX", "ALENDRONATE SODIUM PAROTETINE HCL", "PAROXETENE HYDROCHLORIDE"

"TRAZODONE"

"TRITTICO RETARD", "TRAZOLAN", "DEPRAX", "TRAZODONE", "MESYREL", "TRITTICO", "DEPRAX (R) (TRAZODONE)", "DEPRAX 100", "DEPRAX 100 MG", "TRAZADONE", "DESYREL", "TRAZODONE HCL", "TRAZODONA", "TRAZODON", "TRITICO", "TRAZODONE CAP", "TRAZADON", "TRANZODONA", "TRAZADONA CLORHIDRATO", "TRITTICO (TRAZODONE)", "TRAZODONDE", "TRAZODONE HYDROCHLORIDE", "TRAZIDONE"

"SERTRALINE"

"ZOLOFT", "SERTRALIN", "ASENTRA", "SERLIFT", "AREMIS", "BESITRAN", "ZOLOFT (SERTRALINE)", "SERTRALINE", "SERTRALINE HYDROCHLORIDE", "SETRALINE", "SERTRALINE HCL", "ZOLOFT 50MG", "ZOLOFT 50 MG", "ZOLOFT 25 MG", "ZOLOFT 100MG", "ZOLOFT 75MG", "ZOLOFT 50", "AREMIS 50", "BESITRAN 50", "SERTROLINE", "GEN-SERTRALINE", "NOVO-SERTRALINE", "SERTALINE", "SERTRALINE/ZOLOFT", "ZOLOFT SERTRALINE HCL", "SERLAIN 50 MG", "GLADEM", "SERTRALINA 100", "SELTRA(SERTRALINE)", "ADJUVIN (=SERTRALIN)", "SERTRALINA", "ASERTIN 50 MG", "ASERTIN", "SERTRALINE HCI", "(ZOLOFT) SERTRALINE"

"CITALOPRAM"

"CITALOPRAM", "CELEXA", "LEXAPRO", "CELEXA - CITALOPRAM", "CIPRALEX", "SIPRALEXA", "CYPRALEX", "CIPRAMIL", "CITALOPRAM HYDROBROMIDE", "ESCITALOPRAM", "SEROPRAM", "CETALOPRAM", "SEROPLEX", "ELOPRAM", "ELOPRAM 20MG", "CIPRALEX 10 MG", "CILIFT", "CILIFE", "ESERTIA", "PRISDAL", "PRISDAL 20 MG", "PRISDAL 20", "PRISDAL (TM) 20 MG", "(CITALOPRAM) CELEXA", "GEN-CITALOPRAM", "CELEXQ", "LEXOPRO", "CITALOPRIM", "ESCITALOPRAM OXALATE", "T. CITALOPRAM", "CITALOPRAN", "CITALEC", "SEPRAM", "CITAL", "ZYLORAM", "CIPRALEX ( ESCITALOPRAM)", "LEXAPRO(ESCITALOPRAM)", "CITALEC 20MG TBL.", "SEROPLEX 10", "CITALOPRAM 10 MG", "CITALON", "ENTACT", "CIPRALEX 10", "ESERTIA 20", "SEROPRAM 20 MG", "ECITALOPRAM", "CITALOPRAMI", "LEXAPRO (ESCITOLAPRAM)", "CELEXA (CITALOPRAM)"

### Anti-Anxiety Medications

"LORAZEPAM"

"ORFIDAL", "IDALPREM", "LORAZEPAM", "ATIVAN", "TAVOR", "LORAZEPAN 1 MG", "LORAZEPAN 1MG", "LORAZEPAM (PRN)", "ATIVAN PRN", "TEMESTA EXPIDET", "LORAZEPAN 0.5", "IDALPREM 1 MG", "LORAZEPAM (ATIVAN)", "TEMESTA", "CONTROL LORAZEPAM", "LORENIN", "LORAZEPAN", "KALMALIN", "ATIVAN (EVERY SECOND DAY)", "U-PAN", "LOREZEPAM", "ATIVAN LORAZEPAM"

### Cardiovascular conditions related treatment

"CLOPIDOGREL"

"PLAVIX", "PLAVIX - CLOPIDOGREL BISULFATE", "CLOPIDOGREL", "CLOPIDOGREL HYDROGEN SULPHATE", "CLOPIDOGREL (PLAVIX)", "CLOPIDOGREL BISULPHATE", "PLAYVIX", "CLOPIDROGEL BISULFATE", "PLAVIX 75 MG", "ISCOVER", "PLAVIS", "CLOPIDOGREL BISULFATE"

"ATORVASTATIN"

"LIPITOR", "LIPITOR - ATORVASTATIN CALCIUM", "SORTIS", "TORVACARD", "ATORVASTATIN", "TAHOR", "TAHOR 10", "TOTALIP", "TORVAST 10 MG", "TORVAST 10MG", "TORVAST", "ATORVASTATINE", "ATORVASTATIN (LIPITOR)", "(LIPITOR) ATORVASTATIN", "CARDYL", "CARDYL 20", "PREVENCOR", "CARDYL 10 MG", "CARDYL 10", "CARDYL TM 10 MG", "PREVENCOR 10", "(ATORVASTATIN)", "ATROVASTATIN", "ATORVASTATIN CALCIUM", "ATORVASTATINUM", "ATORVASTATINUM CALCICUM (TORVACARD)", "ATORVASTATINA", "ZARATOR", "ATORIS", "ATORVASTATINA (CARDYL 20)", "CARDYL 40", "ATORVASTATIN CALCIUM(LIPITOR)", "ATORVASTIN CALCIUM"

## Placebos

"VITAMIN B6"

"PYRIDOXINE", "VITAMIN B6", "B-6", "VITAMIN B-6"

"FOLIC ACID" ("VITAMIN B9")

"FOLIC ACID", "MEGAFOL", "MEGAFOL (FOLIC ACID)", "FOLINA", "FOLIN (FOLINA)", "FOLIUMZUUR", "FOLATE", "ACFOL", "FOLIC ACID 1%", "APO-FOLIC ACID", "FOLIC", "FOLATE (FOLIC ACID)", "FOLACIN", "FOLSYRA", "FOLSAN", "FOLSÃ¯Â¿Â¤URE 5 MG", "FOLVITE", "ACID FOLIC SPECIA FOLDINE"

"VITAMIN B12"

"VITAMIN B12", "B-12 INJECTIONS", "B12", "CYANOCOBALAMIN", "VITAMIN B-12", "OPTOVITE B12", "CYTAMEN", "VITAMIN B12 INJECTION", "B12 INJECTIONS", "VITAMIN B12 TABLET", "DOBETIN", "VIT B12", "CYANOCOBALAMIN VITAMIN B12", "B12 VITAMIN", "VIT B12 1ML", "VIT B12 1M", "VIT.B12", "VITAMINE B12 AMPOULES", "OPTOVITE B12 1000", "B12 INJECTION", "B-12", "B 12", "INJECTION Q2W VITAMIN B12", "VITAMIN B12 INJECTION Q2W", "VIT B12 INJ", "VITAMIN B12 INJECTIONS", "VITAMIN B12 INFECTION", "B12 INJ", "V B12", "VITAMINE B12", "CYANACO BALAMIN", "VITAMIN B12 500 MCG", "B-12 INJECTION", "B-12 SHOT", "CYANOCOBALAMINE", "VITAMIN B12 INJ", "CYANOCOBALAM", "VITAMIN B 12", "VITAMIN B12 TABLETS", "B-12 VIT", "CYANOCOBALAMIN ORAL TABLET", "VITAMIN-B12", "B12 500 MG", "B-12 VITAMIN", "BEHEPAN", "VITAMIN B12 (CYANOCOBALAMIN)", "VITAMIN B12 (4 TIMES PER YEAR)", "METHYLCOBAL", "VITAMINE B12 AMPOULE", "B 12 VITAMINE", "VITAMIN B12 INJECTABLE", "B12 VITAMIN INJECTION", "VIT B-12", "VIT. B12", "B -12 VITAMIN", "VIT. B-12", "VIT. B - 12", "B12-VITAMINE", "TIME RELEASE VITAMIN B12"

"VITAMIN C"

"VITAMIN C", "VITAMIN C POWDER", "CEBION 1000MG", "CEBION 1000 3/WEEKS", "CEBION 1000", "ASCORBIC ACID", "VIT C", "VITAMINE C", "V C", "VITAMIN C-COMPLEX", "VITAMIN 'C'", "VITAMIN-C", "VITAMIN C WITH ROSE HIPS", "VITAMIN C WITH ROSEHIPS", "CELASKON", "ASCORBIN", "VIT -C", "VIT-C", "VITAMIN C 500 MG", "VIT. 'C'", "VIT 'C'", "VITAMIC C"

"VITAMIN E"

"VITAMIN E", "VITAMINE", "VIT E", "ALPHA-TOCOPHEROL", "VITAMINE E", "TOCO 500", "TOCOLION", "TOCO", "RIGENTEX", "EPHYNAL", "EPHINAL", "EPHYNAL 300 MG", "VITAMIN E BD", "AUXINA E (MAIN INGREDIENT: TOCOFEROL)", "AUXINA E (R) (VITAMIN E)", "AUXINAE", "AUXINAE 400", "AUXINA E 400", "V E", "VITAMIN 'E'", "NATURAL E 400", "VIT. E", "VITAMIN-E", "VIT E 1000 IU", "VITAMIN E.", "TOCOPHEROLS", "(VITAMIN E)", "TOCOPHEROL", "TOCEPHEROL", "E-MED FORTE", "OPTOVIT E", "OPTOVIT", "TOKOVITAN", "TOCOVITAN", "IDO-E", "VITAMIN E", "SANHELIOS", "SURSUM 400 MG", "SURSUM", "JUVELA", "TOCOPHEROL ACETATE", "VITAMINI E", "VIT. 'E'", "VIT 'E'", "A-TOCOPHERAL", "VITAMIN E (TOCOPHEROL)", "VITAMIN E TOCOPHEROL", "TOCOPHERAL", "TOCOPHEROL (VIT E)", "VIT E 1000 IU BID"

"MULTIVITAMIN"

"MULTIVITAMIN", "VINEYARD BLEND NUTRITIONAL MULTIVITAMIN", "VITASMART MULTIVITAMIN", "MULTI-VITAMIN", "MVI", "MULTI VITAMIN", "MULTIPLE VITAMINS", "MULTIPLE VITAMIN", "MULTIVITAMINS", "MUILTIVITAMIN", "HYPERVITE MULTIVITAMINS", "VITELLE MULTI VITAMIN B", "BIO ACE EXCELL (MULTIVITAMIN)", "HEATHERIES MULTIVITAMINS", "MULTIVITES", "MULTIVIT", "DS 24", "LACTOVITA", "VITA CHOICE (MULTIVITAMIN)", "MVI (MULTI-VITAMINE)", "B12 WITH VITAMINS", "MULTI VITAMINS", "MULTIVITAMINE", "FORMULE FORTE VITAMINS", "VITAVIM", "MILTIVITAMINES", "MULTIVITAMIN A THRU Z", "MULTIVITTAMIN", "MULTI-VIT", "VITAMIN MULTI", "MUTIVITAMIN", "MULT-VITAMIN", "RIGHT CHOICE MULTIVITAMIN AM FORMULA", "RIGHT CHOICE MULTIVITAMIN PM FORMULA", "MVI WITH FOLIC ACID", "KIRKLAND MULTIVITAMIN", "MULTIVITAMINES", "SUPER AYTINAL MULTIVITAMIN", "MULTIVITAMINS AND MINERALS", "SENTURY VITE", "MEGA-VITAMIN", "VITAMINS", "VITAMINS BPC (MULTIVITAMINS)", "BARTELL'S BRAND MULTIPLE VITAMIN", "MULTI VIT.", "METABALANCE 44", "IMMUNPLEX (MULTIVITAMIN)", "MULTI VIT", "MULTI - VITAMIN", "MULTI-VITAMINS", "MULTIVITAMIN W/ MINERALS", "ATKINS BASIC 3 MULTI VITAMIN", "MULTIVITAMIN + MINERALS", "MULTI - VIT", "SENTRY VITE", "MULTIVITAMIN AND MINERAL", "MULTIVITAMIN MINERALS", "MULTIVITAMIN WITH MINERALS", "ACF-223 MULTIVITAMIN", "OMEGA VITAMIN", "MVI-FRUIT PLUS", "WOMEN'S MULTIVITAMIN", "ANTIOXIDANT MULTIVITAMIN"

"FISH OIL"

"FISH OIL", "PRO-OMEGA", "OMEGA 3 FISH OIL", "OMEGA-3", "FISH OIL CAPSULES", "OMEGA 3", "OMEGA FISH OIL", "FISH OIL CAPSULE", "ESKIM", "ESAPENT", "OMEGA 3&6", "FISHOIL", "OMEGA OIL 3", "OMEGA 3 OIL", "OMEGA OIL", "SUPER EPA (40% EPA, 30 DHA WHICH ARE ESSENTIAL FATTY ACIDS. ACTIVE INGREDIENTS:1. FISH OIL 500MG / 2. OMEGA-3 FATTY ACIDS 350MG / 3. EPA-200MG (EICOSAPENTA ENOIC ACID) / 4.DHA-150MG (DOCOSAHEXA EXA EN", "FISH OIL CAP", "FISH OIL OMEGA 3", "OMEGA 3-6-9", "SALMON OIL", "SALMON AND WILD FISH OIL", "OMEGA 369", "OMEGA 3 FATTY ACIDS", "NATURAL FISH OIL", "OMEGA 3 FISH 0.13", "OMEGA 3 FISH OILS", "FISH OIL CONCENTRATE/OMEGA 3", "ULTRA OMEGA FISH OIL DHA/EPA", "FISH OIL CONCENTRATE", "OMEGA COMPLETE", "NATURAL FISH OIL CONCENTRATE", "FISH OILS", "OMEGA-3 FISH OIL CONCENTRATE", "OMEGA 3 FISH OIL CONCENTRATE", "OMEGA 3 ACID", "OMEGA 6 FISH-OIL", "OMEGA 3 FISH-OIL", "FISH OIL TABS", "OMEGA 3 FATTY ACID", "OMEGA FISH OILS", "FISH OIL NON PRESCRIPTION", "OMEGA-3 FATTY ACIDS", "OMEGA FATTY ACID", "OMEGA 3 SYRUP", "OMACOR", "OMEGA-3 FISH OIL", "FISH OIL CAPSULES, CHOLESTEROL FREE", "OMEGA 3 -FISH OIL", "FISH CONCENTRATE", "DEEP SEA FISH OIL", "OMEGA 3 SALMON OIL", "FISH OIL - OMEGA 3", "FISH OIL WITH OMEGA-3"

"GINKGO BILOBA"

"TEBOFORTAN", "TANAKAN", "GINKGO BILOBA", "GINGIO", "TEBONIN", "GINGKGO BILOBA", "GENKO BILOBA", "GINGKO BILOBA", "GINKGO FORTE", "GINGKO BILOBA 7500MG", "GINGKO 7500 NATURE'S OWN", "GINGKO BILOBA FORTE", "GINGKO FORTE", "GINKGO", "GINKO BILOBA", "TANAKAN (GINGKO BILOBA)", "TANAKA GINKO BILOBA", "TAVONIN", "GINKO", "GINGKO BILOBO", "GINGOFORCE", "GINGO BILOBA", "GINGKO", "GINGKOBILOBA", "GINKGO BILBOA", "GINGKO BILBOA", "GINKO BILBOA", "GINKO 2000", "MEMFIT", "BIO-FLOW", "BIOCURE VITAMINE INTELLECT", "GINGKO BILLOBA", "TEBOKAN", "MEMOPLANT", "TEBOKAN /GINGKO BILOBAE/", "GINKGO BILOBAE (TEBOKAN)", "GINKOBILUBA", "BIOBILOBA", "GINKGO BILOBA EX.", "GINKGO.BILOBA EX", "GINEXIN", "GINKGO-BILOBA EXT.", "GINKGO-BILOBA EXT", "GINKORT FORT", "GINGIUM", "TEBONIN 120", "TEBONIN INTENS", "TEBONIN KONZENT", "TEBONIN FORTE", "GINKOCER", "GINGKO BILOBA EXTRACT", "GINGOBIEL", "GINATON", "GINKGO LEAF TABLET", "GINKGO LEAF (A TRADITIONAL CHINESE MEDICINE)", "GINKGOBILOBA", "GINKOBA", "GINKO-BILOBA"

1. Time Frames between MMSE-Tests


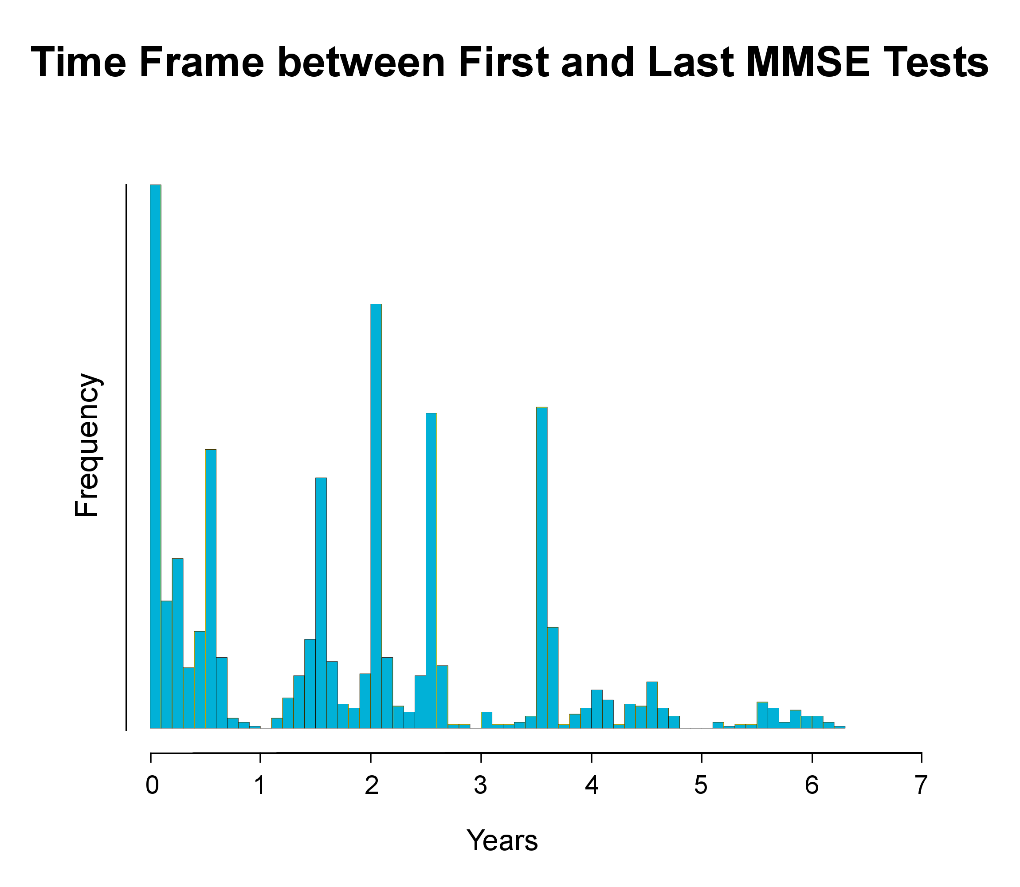


*Figure S8*. **Time frames between the first and last MMSE-tests.** This histogram contains the time frames between the first and last MMSE-test visit dates in the CAMD database.

1. Binomial test p-values calculation for medications associated with transitions across patient groups

Significant associations between medication intakes and patient group transitions with Binomial test *p*-values lower than 0.05 in both training and validation sets are depicted in Table S1.

*Table S1*. **Details on Binomial test *p*-values calculation for medications associated with transitions between patient groups.**

| **Transition Cohort** | | **Comparison Cohort** | | **Treatment** | **Training Set** | | | **Validation Set** | | |
| --- | --- | --- | --- | --- | --- | --- | --- | --- | --- | --- |
| **From** | **To** | **From** | **To** |  | **Rate Tran.** | **Rate Comp.** | ***p*-value** | **Rate Tran.** | **Rate Comp.** | ***p*-value** |
| *C* | *C* | *C* | Union(*C, I, F, D, A*) | Multivitamins | 30/60 (0.5) | 50/138 (0.36) | 3.1·10^-2^ | 33/76 (0.43) | 54/174 (0.31) | 2.5·10^-2^ |
| *I* | *A* | *I* | Union(*C, I, F, D, A*) | Citalopram | 4/10 (0.4) | 14/177 (0.079) | 0.0056 | 4/18 (0.22) | 12/176 (0.068) | 0.031 |
| *D* | *A* | *D* | Union(*C, I, F, D, A*) | Ginkgo Biloba | 6/67 (0.09) | 12/380 (0.032) | 0.019 | 6/59 (0.1) | 14/316 (0.044) | 0.046 |

Significant associations between the medication intake incidence rates and transitions between patient groups, listed in the column “Transition Cohort” (“Tran.”), in relation to transitions specified in the column “Comparison Cohort” (“Comp.”), are provided in this table. Medication titles are listed in the column “Treatment”. “Rate” indicates the number of patients under a particular treatment out of the total number of patients in a given transition cohort; the corresponding ratios calculated based on these values are provided in parenthesis. The Binomial test *p*-values calculated by comparing the medication intake incidence rates between the transitions listed in the columns “Transition Cohort” 'and “Comparison Cohort”, are listed in the corresponding columns (“*p*-value”). Results for the training and validations data sets are listed separately. Abbreviations: *C*: considerate, *I*: inattentive, *F*: forgetful, *D*: distant, *A*: absent.

1. Patient Group Identification in Clinics

In this section we describe how the centroids defined in this study can be employed in clinics. Consider a patient completing a cognitive test with *x_reg_* scores in the category *registration*, *x_att_* in *attention*, and *x_rec_* in *recall*, where the maximal numbers of scores in each category are *m_reg_*, *m_att_* and *m_rec_*, respectively. Thus, the success rates ***s*** of that patient in the categories *registration*, *attention* and *recall* are calculated in accordance with Equation S1.

*Equation S1*. **Success rates calculation.**

$$s=(s_{reg},s_{att},s_{rec})=\left(\frac{x_{reg}}{m_{reg}}\cdot100\%,\frac{x_{att}}{m_{att}}\cdot100\%,\frac{x_{rec}}{m_{rec}}\cdot100\%\right)$$

To define which patient group this particular sample belongs to, a calculation of the Euclidean distance d between this sample and each centroid ***c*** is necessary. This computation is depicted by Equation S2; *c^cognisant^* is a centroid from Table 1 corresponding to the patient group *considerate*, and d*^cognisant^* is the distance between this centroid and the given sample. An analogous calculation is also conducted for the remaining four patient groups to assess the distance of the sample to all groups.

*Equation S2.* **Distance calculation.**

$$\text{d}^{cognisant}=\sqrt{(c_{reg}^{cognisant}-s_{reg})^{2}+(c_{att}^{cognisant}-s_{att})^{2}+(c_{rec}^{cognisant}-s_{rec})^{2}}$$

$$\text{d}^{inattentive}=\sqrt{(c_{reg}^{inattentive}-s_{reg})^{2}+(c_{att}^{inattentive}-s_{att})^{2}+(c_{rec}^{inattentive}-s_{rec})^{2}}$$

$$\text{d}^{forgetful}=\sqrt{(c_{reg}^{forgetful}-s_{reg})^{2}+(c_{att}^{forgetful}-s_{att})^{2}+(c_{rec}^{forgetful}-s_{rec})^{2}}$$

$$\text{d}^{distant}=\sqrt{(c_{reg}^{distant}-s_{reg})^{2}+(c_{att}^{distant}-s_{att})^{2}+(c_{rec}^{distant}-s_{rec})^{2}}$$

$$\text{d}^{absent}=\sqrt{(c_{reg}^{absent}-s_{reg})^{2}+(c_{att}^{absent}-s_{att})^{2}+(c_{rec}^{absent}-s_{rec})^{2}}$$

From the resulting distance values (d*^cognisant^*, d*^innatentive^*, d*^forgetful^*, d*^distant^* and d*^absent^*), the one with the minimal value represents the patient group this particular sample attributes to. Two numeric examples of how to determine a patient group are provided below.

## Example 1

Consider a patient A repeating the names of all three objects in the category *registration* (*x_reg_*=3, *m_reg_*=3 and thus *s_reg_*=3/3·100%=100%), spelling backwards the first four letters of a five-letter word (*x_att_*=4, *m_att_*=5 and thus *s_att_*=4/5·100%=80%) and recalling only one out of the three previously learned and repeated objects (*x_rec_*=1, *m_rec_*=3 and thus *s_rec_*=1/3·100%=33.3%). The Euclidean distance between these results and the centroid corresponding to the *cognisant* patient group is 53.1%, to the *inattentive* 62%, *forgetful* 25.3%, *distant* 64.7%, and *absent* 90.4%, as detailed in Equation S3.

*Equation S3.* **Distance calculation to each group centroid.**

$$d^{cognisant}=\sqrt{\left( 100\%-100\% \right)^{2}{+\left( 98\%-80\% \right)}^{2}{+\left( 83.3\%-33.3\% \right)}^{2}}=53.1\%$$

$$d^{inattentive}=\sqrt{\left( 93.3\%-100\% \right)^{2}{+\left( 44\%-80\% \right)}^{2}{+\left( 83.3\%-33.3\% \right)}^{2}}=62\%$$

$$d^{forgetful}=\sqrt{\left( 93.3\%-100\% \right)^{2}{+\left( 94\%-80\% \right)}^{2}{+\left( 13.3\%-33.3\% \right)}^{2}}=25.3\%$$

$$d^{distant}=\sqrt{\left( 100\%-100\% \right)^{2}{+\left( 21\%-80\% \right)}^{2}{+\left( 6.7\%-33.3\% \right)}^{2}}=64.7\%$$

$$d^{absent}=\sqrt{\left( 43.3\%-100\% \right)^{2}{+\left( 18\%-80\% \right)}^{2}{+\left( 0\%-33.3\% \right)}^{2}}=90.4\%$$

Thus, the patient A clearly belongs to the group *forgetful*, where the distance to the corresponding centroid is half of the length to the next closest patient group, the *cognisant* in this case. Exploiting the results from Table 2, it can be assumed that there is an approximately 15% chance for this patient to become *cognisant* again within the next four years, a 52% chance to retain this status for up to four years, and a 20% chance to decline to *distant* within the same time frame. Furthermore, employing the previously proposed hypothesis on the relation between the disease progression and patient groups, it can be stated that the patient A progressed to a further stage than *cognisant*, with two more phases she/he will probably transit to (*distant* and *absent*).

## Example 2

In another example, consider a patient B passing a non-standard MMSE test, where she/he is able to repeat the names of all four objects presented to her/him (*x_reg_*=4, *m_reg_*=4 and thus *s_reg_*=4/4·100%=100%), is only capable of reproducing the first letter when spelling backwards a six-letter word(*x_att_*=1, *m_att_*=6 and thus *s_att_*=1/6·100%=16.7%) and remembering one out of the four previously repeated and memorized objects (*x_rec_*=1, *m_rec_*=4 and thus *s_rec_*=1/4·100%=25%). The Euclidean distance values to the centroids of the *cognisant*, *inattentive*, *forgetful*, *distant* and *absent* groups are 100%, 64.7%, 78.5%, 18.8% and 62%, respectively (in accordance with Equation S4).

*Equation S4.* **Distance calculation to each group centroid.**

$$d^{cognisant}=\sqrt{\left( 100\%-100\% \right)^{2}{+\left( 98\%-16.7\% \right)}^{2}{+\left( 83.3\%-25\% \right)}^{2}}=100\%$$

$$d^{inattentive}=\sqrt{\left( 93.3\%-100\% \right)^{2}{+\left( 44\%-16.7\% \right)}^{2}{+\left( 83.3\%-25\% \right)}^{2}}=64.7\%$$

$$d^{forgetful}=\sqrt{\left( 93.3\%-100\% \right)^{2}{+\left( 94\%-16.7\% \right)}^{2}{+\left( 13.3\%-25\% \right)}^{2}}=78.5\%$$

$$d^{distant}=\sqrt{\left( 100\%-100\% \right)^{2}{+\left( 21\%-16.7\% \right)}^{2}{+\left( 6.7\%-25\% \right)}^{2}}=18.8\%$$

$$d^{absent}=\sqrt{\left( 43.3\%-100\% \right)^{2}{+\left( 18\%-16.7\% \right)}^{2}{+\left( 0\%-25\% \right)}^{2}}=62\%$$

Thus, the patient B belongs to the *distant* group with a rather poor prognosis: there is only a 3% chance for her/him to improve to *cognisant*, while there is an approximately 57% chance for this patient to retain the *distant* status for up to four following years; the probability of deteriorating to the *absent* group is 18% (Table 2). Prescription of ginkgo biloba for this patient should occur with caution as it may be associated with a cognitive decline to *absent* (Table 4).

1. Patient group identification in clinics: Calculator

We implemented a calculator that can be employed in clinics to assign a patient to a group in accordance with results obtained in this study. It is provided in a separate Excel-file (Table S2).

*Table S2*. **Patient group calculator.** Calculator implemented in Excel can be employed in clinics to assist the assignment of a patient to a group (*cognisant*, *inattentive*, *forgetful*, *distant* or *absent*) based on her/his performance in the cognitive test categories *registration*, *attention* and *recall*.
